# Socioeconomic inequalities in effective service coverage for reproductive, maternal, newborn, and child health: a comparative analysis of 39 low-income and middle-income countries

**DOI:** 10.1016/j.eclinm.2021.101103

**Published:** 2021-09-07

**Authors:** Kanya Anindya, Tiara Marthias, Sukumar Vellakkal, Natalie Carvalho, Rifat Atun, Alison Morgan, Yang Zhao, Emily SG Hulse, Barbara McPake, John Tayu Lee

**Affiliations:** a Nossal Institute for Global Health, The University of Melbourne, Melbourne, Australia; b Department of Public Health, Faculty of Medicine, Public Health and Nursing, Universitas Gadjah Mada, Yogyakarta, Indonesia; c Department of Economic Sciences, Indian Institute of Technology Kanpur, Kalyanpur, Uttar Pradesh, India; d Center for Health Policy, School of Population and Global Health, The University of Melbourne, Melbourne, Australia; e Department of Global Health and Population, Harvard T.H. Chan School of Public Health, Harvard University, Boston, MA, United States; f Global Financing Facility, The World Bank Group, Washington, DC, United States; g WHO Collaborating Centre on Implementation Research for Prevention and Control of Noncommunicable Diseases, Melbourne, VIC, Australia; h The George Institute for Global Health at Peking University Health Science Center, Beijing, China; i Department of Primary Care and Public Health, School of Public Health, Imperial College London, United Kingdom

## Abstract

**Background:**

Reducing socioeconomic inequalities in access to good quality health care is key for countries to achieve Universal Health Coverage. This study aims to assess socioeconomic inequalities in effective coverage of reproductive, maternal, newborn and child health (RMNCH) in low- and middle-income countries (LMICs).

**Methods:**

Using the most recent national health surveys from 39 LMICs (between 2014 and 2018), we calculated coverage indicators using effective coverage care cascade that consists of service contact, crude coverage, quality-adjusted coverage, and user-adherence-adjusted coverage. We quantified wealth-related and education-related inequality using the relative index of inequality, slope index of inequality, and concentration index.

**Findings:**

The quality-adjusted coverage of RMNCH services in 39 countries was substantially lower than service contact, in particular for postnatal care (64 percentage points [pp], *p-*value<0·0001), family planning (48·7 pp, *p*<0·0001), and antenatal care (43·6 pp, *p*<0·0001) outcomes. Upper-middle-income countries had higher effective coverage levels compared with low- and lower-middle-income countries in family planning, antenatal care, delivery care, and postnatal care. Socioeconomic inequalities tend to be wider when using effective coverage measurement compared with crude and service contact measurements. Our findings show that upper-middle-income countries had a lower magnitude of inequality compared with low- and lower-middle-income countries.

**Interpretation:**

Reliance on the average contact coverage tends to underestimate the levels of socioeconomic inequalities for RMNCH service use in LMICs. Hence, the effective coverage measurement using a care cascade approach should be applied. While RMNCH coverages vary considerably across countries, equitable improvement in quality of care is particularly needed for lower-middle-income and low-income countries.

**Funding:**

None.

**Research in context**

**Evidence before this study**

Reducing socioeconomic inequalities in reproductive, maternal, newborn and child health (RMNCH) is key to achieve the health Sustainable Development Goals and other equity-related goals. As of July 25, 2021, a Pubmed search of the terms (“reproductive health” OR "maternal health” OR “neonatal health” OR “child health”) AND (“effective coverage” OR “quality of care”) AND (“inequality” OR “inequity”) retrieved 39 results. Analysis by Arsenault et al., found large differences in the quality of antenatal care (ANC) across socioeconomic groups. However, this study merely focused on ANC and did not apply the effective care cascade framework.

**Added value of this study**

Using the RMNCH effective care cascade framework, we found the crude and quality-adjusted coverage of RMNCH services in all countries included were substantially lower than service contact. Compared with socioeconomic inequalities estimated by quality-adjusted outcomes, contact coverage significantly underestimates the levels of socioeconomic inequalities for effective coverage of RMNCH service use in low- and middle-income countries (LMICs).

**Implications of all the available evidence**

These findings call for the use of measures of socioeconomic inequalities based on service, crude, and quality-adjusted measurements, especially when these measurements are used to inform priority setting and policy decision for the provision of healthcare services to reduce inequalities in effective coverage and health outcomes.

Alt-text: Unlabelled box

## Introduction

Achieving equitable access to reproductive, maternal, newborn, and child health (RMNCH) services is the top priority for international development. The Sustainable Development Goals (SDGs) set ambitious targets to reduce maternal and neonatal mortality to less than 70 per 100,000 live births (target 3.1) and to end preventable deaths of neonatal and children under-five years of age (target 3.2) by 2030 [1]. Despite gradual improvements in the coverage and access to RMNCH services, maternal, newborn and child mortality remain high in low- and middle-income countries (LMICs) [2], which account for 94% of maternal deaths and 78% of under-five deaths [3,4]. Lower socioeconomic groups face higher RMNCH-related morbidity and mortality [2,5], due in part to inadequate access to high-quality RMNCH services [2].

Examination of health system performance in addressing socioeconomic inequalities in RMNCH service use is critical for policy-making and priority setting. Several studies have documented pro-poor inequality in the coverage of RMNCH services [6,7]. According to WHO's State of Inequality report, the richest-to-poorest difference in coverage of skilled birth attendance reached up to 80 percentage points [8]. However, a major limitation of the published studies is the widespread use of service contact, which estimates the percentage of the population of interest who made contact with health service providers for their health condition, to assess the performance of health systems and levels of socioeconomic inequalities [9,10]. While data on contact coverage is more feasible to collect and can typically be estimated from household surveys, it does not take into account effectiveness of service, which is crucial for ensuring improvement in health outcome [11,12].

A useful approach to assess whether patients receive high-quality care is based on the effective coverage measurement [9,11,13], which has been recommended by WHO and UNICEF for health system performance assessment in RMNCH care [14,15]. Effective coverage is defined as ‘the fraction of potential health gain that can be delivered to the population through the health system’[14]. According to the effective coverage approach, a comprehensive assessment of service coverage should incorporate service readiness, completeness, and quality of the service, or health outcomes achieved. Building on Tanahashi's framework [16], Amouzou et al. proposed an effective coverage framework for RMNCH and nutrition to evaluate health-service coverage based on a six-step cascade of care, including service contact, the likelihood of services, crude coverage, quality-adjusted coverage, user-adherence-adjusted coverage, and outcome-adjusted coverage [11]. A major advantage of this approach is that it offers a standardised way of assessing health-system performance, identifying gaps at each step of the coverage cascade, and the loss of potential health benefits. This allows for determining which health systems challenges require immediate attention, for example, the gap between crude and quality-adjusted coverages implies that the necessary practices have not been delivered according to quality-of-care standards. While effective coverage is ideally measured as outcome-adjusted coverage, quality-adjusted coverage also could also serve as a suitable proxy of effective coverage when the outcome measure is arduous to measure [14].

Understanding socioeconomic inequalities in access to effective healthcare services for RMNCH is crucial for designing appropriate evidence-based programmes and policies. To our knowledge, no study has compared estimates of socioeconomic inequalities in RMNCH service use between service contact and quality-adjusted measures, apart from Arsenault et al., which only focused on antenatal care (ANC) coverages [10]. We present the first study of 39 LMICs for the period of 2014–2018, assessing inequalities in coverage and access to effective care for RMNCH. Specifically, we use the effective coverage cascade framework and different indices of inequality to estimate the level of socioeconomic inequality in service contact, crude and quality-adjusted measures across and within these countries.

## Methods

### Sample and data

We used the most recent Demographic and Health Surveys (DHS-7), which were conducted between 2014 and 2018 in 39 countries (17 low-income, 17 lower-middle-income, and five upper-middle-income countries, see Appendix 1). The DHS is a cross-sectional survey that is typically conducted once every five years [17]. The DHS collects information about sociodemographic characteristics and health indicators, with a strong focus on RMNCH. It uses standardised questionnaires to ensure data comparability across countries. Notably, the DHS adopted two-stage cluster sampling design approaches and is nationally representative to make inferences on RMNCH indicators. Procedures and questionnaires for the DHS-7 had been reviewed and approved by the ICF International Institutional Review Board (IRB). The report and dataset are publicly available at https://dhsprogram.com/data/. A detailed description of the sampling strategies and methodology is available elsewhere [18].

We analysed data from the following target groups based on the outcome variables assessed in our study: (1) women of reproductive age (15–49 years) for family planning (FP); (2) women of reproductive age who had a live birth in the three years preceding the survey for the outcomes of ANC, delivery care, and postnatal care (PNC); (3) children age 12–23 months for immunisation coverage; (4) children under five years old for the diarrhoea treatment and the use of insecticide-treated nets (ITNs). After selecting the target groups, we removed those who had missing values in independent variables or covariates (<1% of the pooled sample). The total sample size for each outcome are: 247,232 (FP), 354,605 (ANC and delivery care), 348,051 (PNC), 125,286 (immunisation), 80,572 (diarrhoea treatment) and 188,046 (use of ITNs). Total samples by country and outcome are available in Appendix 2–3. This study adheres to the Strengthening the Reporting of Observational Studies in Epidemiology (STROBE) reporting guidelines.

### Variables

#### Outcomes variables

Our primary measure of interest was the effective coverage of RMNCH services, defined as the proportion of individuals receiving the RMNCH services among those who need it. Building on effective coverage framework proposed by Amouzou et al. [11] seven RMNCH services that are available in the DHS dataset were examined in this study: (1) FP services; (2) ANC; (3) delivery care; (4) PNC; (5) immunisation; (6) diarrhoea treatment; and (7) the use of ITNs (in 14 malaria-endemic countries only).

We applied the effective coverage cascade framework to quantify the use of services at the different conditional stages and to identify gaps at each stage [11]. Starting from the target population, which are those who in need of a service, the four stages include: i) service contact, which is the proportion of target population who visit a health facility for care, ii) crude coverage, which is the proportion of the target population who receive a needed health intervention, iii) quality-adjusted coverage, which is the proportion who receive service according to recommended standards, and iv) user-adherence-adjusted coverage, which is the proportion of the target population who receive recommended standards services and adhere to the treatment guideline**.** Each stage in the cascade is conditional on the preceding stage. Table 1 and Appendix 4–6 outline the detailed definitions and how the coverage measure of the intervention was estimated. Due to several data limitations, some of the outcomes cannot be measured in the full range of stages. For example, the measurements of delivery care and the use of ITNs were only up to the crude coverage as the indicators of service quality were not consistently measured in all countries. Moreover, the quality-adjusted coverage for immunisation could only be reflected by the continuity of inoculations rather than standard-of-care. We also relied on quality-adjusted and user-adherence coverage as proxy measurements of effective coverage because of the absence of outcome data.

**Table 1:** Components of RMNCH effective coverage indicators.

**Intervention**	**Target Population**	**Service contact**	**Crude coverage**	**Quality-adjusted coverage**	**User-adherence adjusted coverage**
Family planning	Women aged 15–49 years old who were sexually active, not currently pregnant, intended to space or limit pregnancy, and have not had sterilisation.	Women who visited the health facility in last 12 months (for any reason) or, women who had been visited by family planning (FP) worker in the last 12 months	Use a modern FP method	Receive information about: - side-effect of the current methods - how to deal with the side effects - other FP methods	N/A
Antenatal visit	Women age 15–49 years old who had given live birth in three years preceding the survey	Use any number of antenatal care (ANC) service from skilled providers (see Appendix 7).	Use at least 4 times ANC service	Use at least 4 times and receive key components of ANCComponents of ANC: - Blood pressure taken - Urine sample taken - Blood sample taken - Given/bough iron tablets/syrup	Receive quality-adjusted ANC service AND adhered to consuming iron tablets for ≥ 90 days during pregnancy
Delivery care	Women age 15–49 years old who had given live birth in three years preceding the survey	Skilled birth attendance (SBA) (see Appendix 7).	SBA at a health facility	N/A	N/A
Postnatal care	Women age 15–49 years old who had recently given live birth in three years preceding the survey	Newborn receive postnatal care (PNC) from a skilled provider. All births attended by SBA were included as having PNC service contact (see Appendix 7).	Receive PNC in the first 24 h after birth or delivery attended by SBA.	Receive PNC in the first 24 h after birth from health providers, weighed, and received BCG vaccination before 1 month	N/A
Immunisation	Children age 12–23 months	Received DPT-1	Receive three doses of DPT-containing vaccines	Receive three doses of DPT-containing vaccines and one dose of measles vaccine	Receive all timely vaccination: - DPT: 2, 4, 6 months - Measles: ≤12 months
Diarrhoea treatment	Children under-five years reported having had diarrhoea in 2 weeks preceding the survey	Seek treatment from a health facility or provider	Receive oral rehydration therapy (ORT) or increased fluids	Receive oral rehydration salt (ORS) mixture	Receive ORS and continued feeding
Use of insecticide-treated nets	Children under-five years in 14 malaria-endemic countries (see Appendix 1)	Owned an insecticide-treated nets (ITN)	Slept under an ITN in the preceding night	N/A	N/A

**Notes:**.

RMNCH — reproductive, maternal, newborn and child health, BCG — Bacille Calmette-Guerin,.

DPT— diphtheria, tetanus toxoid and pertussis.

#### Independent variables

At the country level, we generated dummy variables of three country income groups (low-, lower-middle, and upper-middle-incomes) guided by the World Bank classification of countries by income groups, specific to the survey year [19]. At the individual level, we used the wealth index as an estimate of socioeconomic position. The wealth index was calculated by the DHS using principal component and factor analysis methods based on several indicators, including housing construction materials, type of water access and sanitation facilities, and selected assets. The wealth index was categorised into five quantiles, ranging from Q1 (least wealthy) to Q5 (wealthiest) [18]. As a further independent variable at the individual level, we used the women's educational level (primary or lower, secondary, and tertiary). Moreover, our model also adjusted for women's age at delivery (<20 years, 20–35 years, and 35–49 years), her place of residence (urban, rural), and parity level (≤2 children, >2 children) as covariates [10]. Detailed definitions and categorisations are available in Appendix 4–5.

### Statistical analysis

For the descriptive analysis, we estimated the RMNCH services coverages across effective coverage cascade at country income-level and national-level. Depending on the outcomes, we selected the target population based on eligibility criteria (Table 1) and quantify the coverages across the cascade of care, starting from service contact to user-adherence coverage. The coverage was estimated by dividing the number of individuals who met the criteria at a specific stage by the target population, with each stage is conditional upon the previous stage. Furthermore, we estimated the socioeconomic inequality using simple and complex measures, as suggested by WHO [20]. The simple measures, including difference and ratio, were used to make a pairwise comparison of RMNCH services coverages between the most advantaged (wealthiest, most educated) and least advantaged (least wealthy, least aducated) groups [20]. Subsequently, we calculated complex measures of inequality using the relative index of inequality (RII) that has been widely applied in previous studies [21]. The main strength of complex measures is that the calculations are adjusted for variation in the mean value of the outcome of interest and enables comparing the magnitude of inequalities by accounting for the distribution of outcome variable across the entire socioeconomic groups [22]. We fitted a Poisson regression model with log-link function to generate RIIs which has been recommended by previous studies when the outcomes are not rare [23,24]. We transformed the wealth index into relative rank, namely a ridit-score, scaled from zero to one by arranging the groups in order from lowest to highest socioeconomic position and assigning the cumulative proportion of the population to each group [22]. A coefficient of RII greater than one (RII>1) represents higher inequality, where the most advantaged are more likely to receive services compared with the least advantaged counterparts. Our estimates of the RII models were adjusted for covariates. Detailed measurements of simple and complex measures are available in Appendix 7.

We also estimated other complex measures for inequality, slope index of inequality (SII) and the Erreygers’ normalised concentration index (CI), in the sensitivity analysis. SII indicates the absolute difference in the RMNCH services coverage between the most advantaged and the least advantaged groups by taking into consideration all other subgroups. We used a Poisson regression model with an identity-link function to estimate the differences. CI indicates the extent to which the RMNCH services are concentrated among the higher and lower socioeconomic groups. Erreygers’ normalised CI is preferable over the relative CI as the outcome variables are binary. For both measurements, a positive coefficient (SII or CIX>0) indicates a higher concentration of coverage among the higher socioeconomic group. Multicollinearity was tested using variance inflation factor (VIF). The highest VIF was 2.89 (parity level), indicating that the assumption of reasonable independence among predictor variables was met.

All analyses were conducted using weight, clustering and stratification, using Stata ‘*svyset’* command, to account for multistage sampling design of DHS. As per the DHS manual, we denormalised individual-weight according to the Population Division of the United Nations to calculate the appropriate weight for pooled data [25]. All statistical analyses were carried out using Stata 14.2 SE (Stata Corp., College Station, Texas).

### Role of funding source

There was no funding for this study.

## Results

### Sample characteristics

Three countries with the largest sample sizes are India (25,896 for FP, 135,357 for ANC, delivery care, and PNC, 49,284 for diarrhoea treatment, 22,500 for immunisation), Nigeria (12,422 for FP, 17,302 for ANC, delivery care, and PNC, 6059 for immunisation, 3956 for diarrhoea treatment, 30,713 for ITNs) and Afghanistan (13,846 for FP, 16,121 for ANC, delivery care, and PNC, 5820 for diarrhoea treatment, 7990 for immunisation), and Indonesia (19,706 for FP, 10,000 for ANC, delivery care, and PNC, 3535 for diarrhoea treatment, 2440 for immunisation) (see Appendix 2).

### RMNCH effective coverage cascade in 39 LMICs

**Fig. 1:**
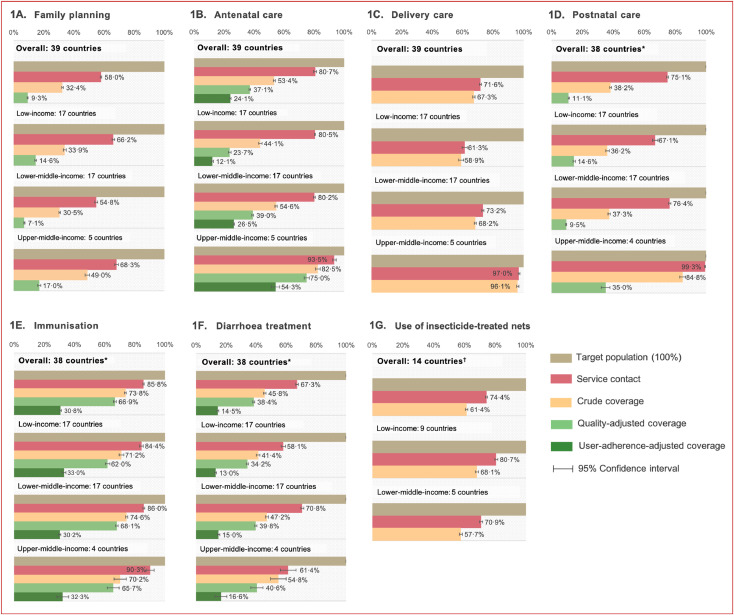
Coverage of RMNCH services, by country income groups. Notes: RMNCH — reproductive, maternal, newborn and child health. List of countries is available in Appendix 1. *38 countries: all countries except Colombia. ^†^14 countries: malaria endemic country only

The overall cascade of care analysis revealed the most substantial loss to effective RMNCH coverage at the stage of service contact. The difference between the population in need (target population) and those who received the service (service contact) was 26·7 percentage points (pp) on average, with 42·0 pp for FP, 19·3 pp for ANC, 28·4 pp for delivery care, 24·9 pp for PNC, 14·2 pp for immunisation, 32·7 pp for diarrhoea treatment, and 25·6 pp for the use of ITNs (Fig. 1). There was a modest loss between the service contact and crude coverage, at 20·1 pp on average, ranging from 4·3 pp for delivery care to 36·9 pp for PNC. Furthermore, on average, there was a 16·2 pp difference between crude coverage and quality-adjusted coverage, ranging from 6·9 pp for immunisation to 27·1 pp for PNC. The marked reduction between the service contact and quality-adjusted coverage is seen in PNC (64·0 pp), FP (48·7 pp), and ANC (43·6 pp). Finally, The average loss between quality-adjusted coverage and user-adherence-adjusted coverage of ANC, immunisation, and diarrhoea treatment was 24·2 pp, with ANC experiencing the slightest decrease (12·7 pp) and immunisation the greatest fall (36·1 pp).

#### Variation in effective RMNCH service coverage by country income groups

Fig. 1 presents the coverage of RMNCH services across countries based on the World Bank country income classification [19]. Our results suggest that upper-middle-income countries had higher service contact, crude, quality-adjusted, and user-adherence-adjusted coverage compared with low- and lower-middle-income in all reproductive, maternal and neonatal health outcomes. For example, for ANC visits, the crude coverage of ANC visits, or women receiving at least four ANC visits, was much higher in upper-middle-income countries (82·5%, 95% CI 80·9–84·1) compared with lower-middle (54·6%, 53·9–55·3) and low-income countries (44·1%, 43·0–45·1). Similarly, there was 75·0% (73·3–76·7) of women who received quality-adjusted ANC visits in upper-middle-income countries, but the proportion was only 39·0% (38·4–39·6) and 23·7% (22·8–24·5) in lower-middle- and low-income countries, respectively.

These indicate that in upper-middle-income countries, almost 90·9% of women with at least four ANC visits received all components of care (82·5% vs 75·0%), whereas only 53·7% of women with at least four ANC visits in low-income countries received all components of care (44·1% vs 23·7%). In terms of immunisation and diarrhoea treatment, the coverages were comparable across all income categories. Detailed figures for each country and income group are presented in Appendix 8–9.

#### Socioeconomic inequalities by country income groups

Table 2 presents the absolute (difference) and relative (ratio) inequality of effective RMNCH services based on simple measures. For most RMNCH services, the wealthiest and most educated group reported greater coverage compared with the least wealthy and least educated group (diff>0 or ratio>1). The pro-rich and pro-educated inequalities were markedly high in ANC, delivery care, and PNC outcomes. The widest inequalities were more pronounced in low- and lower-middle-income countries compared with upper-middle-income countries. However, based on wealth index, upper-middle-income countries had greater inequalities of diarrhoea treatment compared to other income groups. Results of each country are available in Appendix 10–11.

**Table 2:** Simple measures of socioeconomic inequality, by country income groups.




**Notes:**.

CI — confidence interval.

Difference (Diff) =% coverage in most advantaged (wealthiest/tertiary education) –% coverage in most disadvantaged (least wealthy/primary or lower education).

Diff > 0 indicates most advantaged group reported higher coverage of RMNCH services.

Ratio =% coverage in most advantaged /% coverage in most disadvantaged. Ratio > 1 indicates most advantaged group were more likely to receive the RMNCH services.

Dark green indicates smaller inequality, red indicates higher inequality, grey indicates data not available/applicable.

Table 3 shows the relative inequality of effective RMNCH services using complex measurement. Consistent with using simple measures, the RII estimates indicate inequality favouring high socioeconomic groups (RII>1) in all outcomes across the countries examined. Socioeconomic inequalities were more pronounced in lower-income and lower-middle-income countries compared with upper-middle-income countries. Lower-middle-income countries had higher levels of inequalities than low-income countries, except for the use of ITNs. For example, there was no statistically significant difference in the probability of receiving quality-adjusted PNC between the least wealthy and the wealthiest groups in upper-middle-income countries (RII 1·24, 95% CI 0·95–1·63). However, the distribution of quality-adjusted PNC was pro-rich in low-income countries (RII 1·57, 1·37–1·80) and even more inequitably distributed among those who lived in lower-middle-income countries (RII 4·46, 3·99–4·98). We found a similar pattern of inequalities when the measurement was based on women's educational levels (Appendix 12), except for diarrhoea treatments. The greatest wealth-related inequality of diarrhoea treatment was found in upper-middle-income countries (RII>1·5), while according to educational level, the level of inequality was not statistically significant in upper-middle-income countries.

**Table 3:** Complex measures of socioeconomic inequality, by country income groups.

**Outcomes**	**Overall**	**Low-income countries**	**Lower-middle-income countries**	**Upper-middle-income countries**
	RII (95% CI)	RII (95% CI)	RII (95% CI)	RII (95% CI)

**Family Planning**				
Contact	1·12 (1·07–1·16)	1·14 (1·09–1·20)	1·10 (1·05–1·17)	1·10 (1·02–1·19)
Crude	1·21 (1·14–1·28)	1·46 (1·32–1·61)	1·20 (1·12–1·29)	1·06 (0·93–1·20)
Quality	1·35 (1·21–1·50)	1·31 (1·13–1·51)	1·37 (1·18–1·58)	1·11 (0·83–1·48)
**ANC**				
Contact	1·48 (1·45–1·51)	1·14 (1·10–1·19)	1·62 (1·58–1·65)	1·07 (1·01–1·13)
Crude	2·21 (2·13–2·29)	1·65 (1·53–1·79)	2·57 (2·46–2·67)	1·21 (1·10–1·33)
Quality	3·27 (3·11–3·43)	2·59 (2·32–2·89)	4·06 (3·85–4·29)	1·27 (1·14–1·43)
User-adherence	4·07 (3·77–4·35)	2·13 (1·83–2·48)	5·43 (5·05–5·82)	1·22 (0·99–1·49)
**Delivery**				
Contact	1·75 (1·71–1·80)	1·77 (1·65–1·91)	1·86 (1·80–1·91)	1·05 (1·03–1·08)
Crude	1·89 (1·84–1·95)	1·82 (1·69–1·96)	2·03 (1·97–2·10)	1·07 (1·03–1·10)
PNC*				
Contact	1·63 (1·58–1·67)	1·52 (1·42–1·63)	1·71 (1·67–1·76)	1·01 (0·99–1·02)
Crude	1·93 (1·82–2·05)	1·41 (1·28–1·56)	2·30 (2·15–2·46)	1·1 (1·01–1·21)
Quality	3·01 (2·75–3·29)	1·57 (1·37–1·80)	4·46 (3·99–4·98)	1·24 (0·95–1·63)
Immunisation*				
Contact	1·27 (1·24–1·30)	1·14 (1·08–1·19)	1·32 (1·28–1·35)	1·08 (0·96–1·20)
Crude	1·49 (1·44–1·53)	1·24 (1·15–1·33)	1·56 (1·51–1·62)	1·02 (0·81–1·29)
Quality	1·61 (1·56–1·67)	1·28 (1·18–1·39)	1·72 (1·65–1·79)	1·05 (0·82–1·33)
User-adherence	2·4 (2·22–2·59)	1·5 (1·32–1·71)	2·73 (2·49–2·99)	1·3 (0·83–2·04)
Diarrhoea treatment*				
Contact	1·14 (1·09–1·19)	1·11 (1·03–1·20)	1·16 (1·10–1·22)	1·51 (1·10–2·06)
Crude	1·28 (1·19–1·38)	1·19 (1·07–1·32)	1·33 (1·21–1·45)	1·58 (1·11–2·26)
Quality	1·25 (1·15–1·35)	1·13 (1·00–1·28)	1·29 (1·17–1·43)	2·07 (1·24–3·45)
User-adherence	1·35 (1·16–1·58)	1·15 (0·94–1·40)	1·43 (1·18–1·74)	2·65 (1·16–6·06)
**Use of insecticide-treated nets^†^**				
Contact	1·09 (1·05–1·13)	1·21 (1·16–1·26)	0·98 (0·92–1·03)	N/A
Crude	1·00 (0·95–1·05)	1·23 (1·17–1·30)	0·83 (0·77–0·89)	N/A

**Notes:**.

RII — relative index of inequality, CI — confidence interval, ANC — Antenatal care, PNC — Postnatal care.

RII was estimated based on wealth index

*38 countries: all countries except Colombia

†14 countries: malaria endemic countries only (see Appendix 1).

Table 4 shows the estimates of RII by countries. The RII estimates for crude/quality-adjusted coverage in seven outcomes ranged from: (1) FP: 0·41 (95% CI 0·29–0·58) in Nepal to 170·6 (37·75–770·9) in Angola; (2) ANC: 0·87 (0·72–1·04) in Maldives to 19·2 (12·89–28·63) in Bangladesh; (3) delivery care: 0·95 (0·91–1·00) in Maldives to 8·18 (6·97–8·93) in Nigeria; (4) PNC: 0·45 (0·19–1·05) in Guatemala to 487·5 (28·40–8367) in Ethiopia; (5) immunisation: 0·76 (0·59–0·98) in Armenia to 4·77 (3·28–6·93) in Angola; (6) diarrhoea treatment: 0·47 (0·25–0·88) in Lesotho to 2·43 (1·79–3·31) in Guinea; (7) the use of ITNs: 0·65 (0·59–0·72) in Nigeria to 2·07 (1·85–2·33) in Burundi. Inequalities, according to women's educational levels, were similar to those based on the wealth index (Appendix 13). Greater RII estimates were found in Angola, Ethiopia, and Nigeria, while Jordan, Maldives, and South Africa had lower RII compared with other countries.

**Table 4:** Inequality of RMNCH service contact and quality, by socioeconomic status.

**Notes:**.

RII — relative index of inequality, CI — confidence interval.

RII > 1 indicates higher inequality (wealthier groups are more likely to receive services compared to less wealthy groups).

Dark green indicates smaller inequality, red indicates higher inequality, grey indicates data not available/applicable.

#### Comparative inequalities estimate between contact and quality-adjusted measures

We compared the level of inequalities estimated using service contact, crude, quality-adjusted, and user-adherence measures (Table 3). Our results indicate that estimates of socioeconomic inequalities were more pronounced at the higher stages of the effective coverage cascade. For instance, in lower-middle-income countries, the RII estimates of ANC were 2·44 points higher for quality-adjusted coverage (RII 4·06, 95% CI 3·85–4·29) than the service contact coverage (RII 1·62, 1·58–1·65). Compared with lower-middle-income countries, the differences of RII between the quality-adjusted and service contact coverages of ANC were lower in upper-middle-income (quality-adjusted RII 1·27, 1·14–1·43 vs service contact RII 1.07, 1·01–1·13) and low-income countries (quality-adjusted RII 2·59, 2·32–2·89 vs service contact RII 1·14, 1·10–1·19). The difference in the RII estimates between crude coverage and service contact was narrower compared with the difference between quality-adjusted coverage and service contact. In particular, for delivery care and the use of ITNs, the differences were close to zero in all the country income groups.

#### Robustness check

Using SII and concentration index, we found a similar pattern of inequality favouring the wealthiest group in low-income and lower-middle-income countries. The SII estimates show a high inequality level of the ANC, delivery care, and PNC coverages between the wealthiest and least wealthy groups in low-income and lower-middle-income countries (SII>0) (Appendix 14). The strongest absolute socioeconomic inequality for delivery care was seen in Cameroon, for both contact (SII 0·78, 95% CI 0·69–0·90) and crude coverage (SII 0·77, 0·66–0·87). Consistent with RII estimates, some low-income (Afghanistan, Haiti, and Ethiopia) and lower-middle-income countries (Bangladesh, Nigeria, and Kenya) reported high levels of inequalities, with the magnitude of SII higher than 0·50. Similarly, the concentration indexes were mostly positive (CIX>0) in low- and lower-middle-income countries, indicating a higher coverage of RMNCH services among the wealthiest groups (Appendix 15). However, we found relatively small differences in SII estimates between contact and crude/quality-adjusted coverages.

## Discussion

This study systematically estimated levels of socioeconomic inequalities in effective service coverage for RMNCH across 39 LMICs, using the effective coverage cascade framework. We present new findings on the large level of inequalities across and within countries in effective coverage of RMNCH services.

In general, upper-middle-income countries had higher crude, quality-adjusted, and user-adherence-adjusted coverage compared with low- and lower-middle-income in all outcomes, except for crude and quality-adjusted immunisation and service contact of diarrhoea treatment. The largest loss to effective RMNCH coverage was at the stage between the target population and service contact, which indicates the lack of access to healthcare. However, there were substantial gaps between crude and quality-adjusted coverages in FP (23·1 pp), ANC (16·3 pp), and PNC (27·1 pp). The gaps reveal the missing opportunities in delivering health gains to the population due to the low quality of care [26], which calls for the importance of quality improvement.

Our findings reveal that socioeconomic inequalities are particularly evident in lower-middle-income countries. This is aligned with previous evidence which found a narrower gap between the wealthiest and the least wealthy when the national coverage is either very low or very high [27,28]. In countries with moderate coverage of services, i.e., lower-middle-income groups, the wealthiest group will initially pick up the services, resulting in substantially greater coverage and inequality compared to the least wealthy group. While in low-income countries, which generally have poor coverages, the services may not available in both wealthy and least wealthy groups, resulting in smaller socioeconomic inequality [28].

We also found that the estimates of socioeconomic inequalities using service contact measure generally underestimated the level of inequalities compared with crude, quality, and user-adherence-adjusted measures. This result indicates that the assessment of health system performance also considers the timeliness and completeness of the services received relative to the guidelines. Therefore, our study highlights the need for more analysis of the levels of inequalities that goes beyond the bottlenecks in access, but also the bottlenecks in effective coverage in ensuring the timeliness and completeness of RMNCH services in LMICs.

Generally, physical access to ANC and immunisation, is not a large problem for most LMICs, with greater than 80% of service contact coverages in all income groups. However, these LMICs have relatively low coverage of several RMNCH interventions, especially for FP services and diarrhoea treatment. Moreover, the quality-adjusted coverage of RMNCH services remains low, indicating that individuals are not receiving the maximum possible health gains from existing health services [14]. Our findings are consistent with several previous studies from Kenya, Mexico and other LMICs [11,12,29], even for effective coverage of RMNCH services [26,29,30].

Despite a growing body of evidence suggesting that substantial progress has been made in the coverage of RMNCH services in the past decades, large demographic and socioeconomic inequities still remain in most LMICs when considering effective coverage [30]. Maternal health indicators are particularly prone to such inequalities, with the rich-poor ratio reaching over fourfold in some countries [30,31]. At the dawn of the SDGs, progress in the coverage of RMNCH remains insufficient at the national level and across equity dimensions to accelerate towards Universal Health Coverage (UHC) by 2030 [32,33].

The large gap between crude and quality-adjusted coverage indicates a need to focus on interventions that consider the effectiveness of the RMNCH service [32]. To achieve this, the first step is to promote the global use of indicators that could capture the quality aspect of RMNCH services and move away from using the measure of service contact [11]. WHO quality of care framework for RMNCH underlines the importance for countries to use information pertaining to the quality of care to monitor the progress towards UHC and other health-related SDG targets [15]. However, several studies have documented the challenges of quality of care measurement using household-survey data, such as mothers’ inaccuracy recall interventions after delivery (e.g., whether the newborn was dried) and difficulty to recognise the types and quantities of fluids for diarrhoea treatment. On the other hand, incompleteness and inaccuracy of routinely collected data in low-resource settings lead to failure to adequately capture the quality of care [34,35]. Continuous efforts should be made by countries to better document the service provision in the routinely collected health services and thus, could provide an alternative data source to complement findings from household-survey [14]. Combination of multiple data sources would provide more complete information on all stages of the effective coverage cascade. Furthermore, we also stress the importance of improving existing tools by adjusting and harmonizing the data-collection instrument with the global framework and standards to produce the relevant information on the service quality.

The persistent socioeconomic inequality calls for interventions targeting the least wealthy groups in countries with higher coverage levels, such as in upper-middle-income countries. However, countries with far lower coverage than others, such as Afghanistan, Chad, Ethiopia, and Guinea, need to make greater progress in achieving the target of 80% essential health services coverage in 2030. Thus, a whole-population approach may be more suitable in this context. Countries should identify specific barriers to accessing high-quality healthcare services among the target populations, including social and structural barriers, and introduce policies to address them. Financial barriers, for example, could be addressed by expanding pro-poor health insurance coverage. Such interventions have been shown to improve access RMNCH services among the poorer wealth quintiles as well as rural population [36]. Complementary financing programs should also be implemented to address patient referral-associated costs. Supply-side interventions that are contextualised to local needs and resources, such as Colombia's “Salud a su casa” have also shown to contribute to reducing socioeconomic inequalities in maternal and child mortality [37,38].

This study has several caveats. First, to maintain comparability across countries, some quality performance indicators could not be included. For instance, the quality-adjusted ANC visit was only assessed using four clinical components of ANC visits which were consistently collected across countries. For delivery care and use of ITNs, we could only include service contact and crude coverage due to limited information collected by the DHS. Furthermore, our measurement of the quality of care did not adjust for facility readiness and service provision that may further reduce the estimate. Thus, the quality coverage estimates only reflected the minimum conditions required for judging the quality of care and may lead to overestimation. Several quality assessments also could not be ascertained from the DHS data. For instance, the information on the standards in providing child immunisation was not available. And thus, quality-adjusted coverage for immunisation could only be reflected by the continuity of inoculations. Furthermore, our models were controlled for limited independent variables due to inconsistency of certain variables collected between countries in the DHS datasets. Second, DHS is based on self-reporting and recall bias could affect the reported measures of the different services received by women and children. We attempted to minimise the recall bias by limiting our population of interest to women who had live births in the last three years for the ANC, delivery care, and PNC; children aged 12–23 months for immunisation; and children with symptoms of diarrhoea in the last two weeks preceding the survey. Lastly, DHS-7 was conducted over four years, which may limit the comparability across countries.

In conclusion, the reliance on contact coverage significantly underestimates the levels of socioeconomic inequalities for RMNCH service in LMICs. Our findings make a strong case for clearer evidence on socioeconomic inequalities using measures such as effective coverage of RMNCH services that consider the quality of care patients received, to inform priority setting and policy decision for provision of healthcare services to reduce pervasive inequalities in RMNCH services and health outcomes.

## Funding

There was no funding for this study.

## Contributors

KA, TM, and JT conceived and designed the study. KA and TM had access and accept full responsibility for all data associated with this study. KA and TM did the analyses, with input from SV, NC, AM, RA, and JT. KA, TM, SV, YZ, and JT wrote the first draft of the report. SV, NC, AM, YZ, RA, and BM provided critical input in revising the manuscript. EH assisted in drafting the discussion section and proofread all section. All authors contributed to writing, reviewing, and editing multiple versions of the manuscript.

## Data sharing statement

All the individual participant data and statistical analysis plan that underlie the results reported in this article are available for researchers who have submitted an abstract and analysis plan through https://dhsprogram.com/data/. In order to gain access, data requestors will need to sign an online data access agreement.

## Declaration of Competing Interest

We declare no competing interests.

## Appendix Supplementary materials

Image, application 1
