# Supplementary material for: Socioeconomic inequalities in effective service coverage for reproductive, maternal, newborn, and child health: a comparative analysis of 39 low-income and middle-income countries
Source: eClinicalMedicine. 2021 Sep 7;40:101103. doi: 10.1016/j.eclinm.2021.101103 (PMC8430373; doi:10.1016/j.eclinm.2021.101103)
Supplement: Supplementary file 1 [file mmc1.docx]

**Socioeconomic inequalities in effective service coverage for reproductive, maternal, newborn, and child health: a comparative analysis of 39 low-income and middle-income countries**

Appendix 1. List of countries

Appendix 2. Total samples by countries

Appendix 3. Respondents’ background characteristics

Appendix 4. List of variables

Appendix 5. Effective coverage indicators in the previous studies

Appendix 6. List of skilled providers by countries

Appendix 7. The measurement of socioeconomic inequalities

Appendix 8. Coverage by income groups

Appendix 9. Coverage of RMNCH services by country

Appendix 10. Simple measures of socioeconomic inequality based on wealth index

Appendix 11. Simple measures of socioeconomic related inequality based on educational attainment, by countries

Appendix 12. Socioeconomic inequality of RMNCH services based on women’s educational attainment, by income group

Appendix 13. Relative index of inequality of RMNCH services, by educational attainment

Appendix 14. Slope index of inequality of RMNCH services, by socioeconomic status

Appendix 15. Erreygers’ concentration index of RMNCH services

**APPENDICES**

**Appendix 1. List of countries**

| **Countries** | **DHS Year** | **Income group specific to DHS year^[[1]](#footnote-1)^** | **GDP DHS Year adjusted PPP^1^** | **Region** | **Malaria^[[2]](#footnote-2)^** |
| --- | --- | --- | --- | --- | --- |
| Afghanistan | 2015 | Low Income | 2212·8 | South Asia | Low |
| Albania | 2017 | Upper-middle income | 13037·0 | Europe & Central Asia | Free |
| Armenia | 2016 | Lower-middle income | 11303·1 | Europe & Central Asia | Free |
| Angola | 2015 | Upper-middle income | 8036·4 | Sub-saharan Africa | High |
| Bangladesh | 2014 | Lower-middle income | 3511·6 | South Asia | Low |
| Benin | 2017 | Low Income | 3044·5 | Sub-saharan Africa | High |
| Burundi | 2016 | Low Income | 794·6 | Sub-saharan Africa | High |
| Cameroon | 2018 | Lower-middle income | 3603·5 | Sub-saharan Africa | High |
| Colombia | 2015 | Upper-middle income | 14237·6 | Latin America & Caribbean | Low |
| Ethiopia | 2016 | Low Income | 1894·9 | Sub-saharan Africa | Low |
| Ghana | 2014 | Lower-middle income | 4675·8 | Sub-saharan Africa | High |
| Guinea | 2018 | Low Income | 2498·4 | Sub-saharan Africa | High |
| Guatemala | 2015 | Lower-middle income | 8125·4 | Latin America & Caribbean | Low |
| Haiti | 2016 | Low Income | 1766·1 | Latin America & Caribbean | Low |
| India | 2015 | Lower-middle income | 5454·3 | South Asia | Low |
| Indonesia | 2017 | Lower-middle income | 10935·8 | East Asia & Pacific | Low |
| Jordan | 2017 | Upper-middle income | 9841·2 | Middle East & North Africa | Free |
| Kenya | 2014 | Lower-middle income | 3709·2 | Sub-saharan Africa | High |
| Cambodia | 2014 | Low Income | 3364·3 | East Asia & Pacific | Low |
| Lesotho | 2014 | Lower-middle income | 2677·0 | Sub-saharan Africa | Free |
| Mali | 2018 | Low Income | 2283·1 | Sub-saharan Africa | High |
| Myanmar | 2016 | Lower-middle income | 4482·9 | East Asia & Pacific | Low |
| Maldives | 2016 | Upper-middle income | 17582·7 | South Asia | Free |
| Malawi | 2015 | Low Income | 1027·2 | Sub-saharan Africa | High |
| Nigeria | 2018 | Lower-middle income | 5155·1 | Sub-saharan Africa | High |
| Nepal | 2016 | Low Income | 2902·2 | South Asia | Low |
| Papua New Guinea | 2017 | Lower-middle income | 4353·3 | East Asia & Pacific | Low |
| Philippines | 2017 | Lower-middle income | 8120·8 | East Asia & Pacific | Low |
| Pakistan | 2017 | Lower-middle income | 4571·4 | South Asia | Low |
| Rwanda | 2015 | Low Income | 1888·8 | Sub-saharan Africa | Low |
| Senegal | 2017 | Low Income | 3203·9 | Sub-saharan Africa | Low |
| Chad | 2014 | Low Income | 1866·3 | Sub-saharan Africa | High |
| Tajikistan | 2017 | Low Income | 3089·5 | Europe & Central Asia | Free |
| Timor-Leste | 2016 | Lower-middle income | 3355·5 | East Asia & Pacific | Low |
| Tanzania | 2015 | Low Income | 2354·1 | Sub-saharan Africa | High |
| Uganda | 2016 | Low Income | 2073·3 | Sub-saharan Africa | High |
| South Africa | 2016 | Upper-middle income | 12703·8 | Sub-re05saharan Africa | Low |
| Zambia | 2018 | Lower-middle income | 3521·5 | Sub-saharan Africa | High |
| Zimbabwe | 2015 | Low Income | 2958·2 | Sub-saharan Africa | Low |

GDP — Gross domestic product, PPP — Purchasing power parity

**Appendix 2. Total samples by countries**

| **Countries** | | **Outcomes** | | | | | | |
| --- | --- | --- | --- | --- | --- | --- | --- | --- |
|  |  | FP^1^ | ANC^2^ | Delivery care^2^ | PNC^2^ | Immunisation^3^ | Diarrhoea treatment^4^ | Insecticide-treated nets^5^ |
| **Low-income countries** | | |  |  |  |  |  |  |
|  | Afghanistan | 13846 | 16121 | 16121 | 16121 | 5820 | 7990 | N/A |
|  | Benin | 4566 | 7239 | 7239 | 7239 | 2522 | 1342 | 12651 |
|  | Burundi | 5784 | 6995 | 6995 | 6995 | 2596 | 2664 | 12472 |
|  | Cambodia | 7186 | 4077 | 4077 | 4077 | 1441 | 855 | N/A |
|  | Chad | 5110 | 9088 | 9088 | 9088 | 2880 | 3292 | 16901 |
|  | Ethiopia | 4746 | 5541 | 5541 | 5541 | 1929 | 1090 | N/A |
|  | Guinea | 1947 | 4152 | 4152 | 4152 | 1408 | 1011 | 7273 |
|  | Haiti | 5018 | 3572 | 3572 | 3572 | 1196 | 1235 | N/A |
|  | Malawi | 8694 | 9572 | 9572 | 9572 | 3248 | 3402 | N/A |
|  | Mali | 3323 | 5148 | 5148 | 5148 | 1946 | 1590 | 16462 |
|  | Nepal | 3686 | 2761 | 2761 | 2761 | 1025 | 336 | 9275 |
|  | Rwanda | 4964 | 4416 | 4416 | 4416 | 1537 | 905 | N/A |
|  | Senegal | 3995 | 6525 | 6525 | 6525 | 2390 | 2211 | N/A |
|  | Tajikistan | 3346 | 3166 | 3166 | 3166 | 1297 | 802 | N/A |
|  | Tanzania | 4296 | 5558 | 5558 | 5558 | 2158 | 1125 | 9713 |
|  | Uganda | 6259 | 8008 | 8008 | 8008 | 2922 | 2923 | 14710 |
|  | Zimbabwe | 3677 | 3411 | 3411 | 3411 | 1151 | 931 | N/A |
| **Lower-middle-income countries** | | |  |  |  |  |  |  |
|  | Angola | 4727 | 7452 | 7452 | 7452 | 2845 | 1891 | 13619 |
|  | Armenia | 2698 | 949 | 949 | 949 | 345 | 68 | N/A |
|  | Bangladesh | 10296 | 4494 | 4494 | 4494 | 1557 | 371 | N/A |
|  | Cameroon | 3745 | 5034 | 5034 | 5034 | 1824 | 1104 | 9085 |
|  | Ghana | 2539 | 3242 | 3242 | 3242 | 1128 | 671 | 5595 |
|  | Guatemala | 6947 | 6747 | 6747 | 6747 | 2408 | 2239 | N/A |
|  | India | 25896 | 135357 | 135357 | 135357 | 49284 | 22500 | N/A |
|  | Indonesia | 19706 | 10000 | 10000 | 10000 | 3535 | 2440 | N/A |
|  | Kenya | 5386 | 11005 | 11005 | 11005 | 4052 | 2953 | 20093 |
|  | Lesotho | 1919 | 1924 | 1924 | 1924 | 655 | 328 | N/A |
|  | Myanmar | 4215 | 2628 | 2628 | 2628 | 915 | 550 | N/A |
|  | Nigeria | 12422 | 17302 | 17302 | 17302 | 6059 | 3956 | 30713 |
|  | Pakistan | 5682 | 6276 | 6276 | 6276 | 2314 | 2107 | N/A |
|  | Papua New Guinea | 4191 | 5014 | 5014 | 5014 | 1816 | 1313 | N/A |
|  | Philippines | 8077 | 5425 | 5425 | 5425 | 1986 | 652 | N/A |
|  | Timor Leste | 3940 | 3745 | 3745 | 3745 | 1423 | 700 | N/A |
|  | Zambia | 4952 | 5534 | 5534 | 5534 | 1928 | 1432 | 9484 |
| **Upper-middle-income countries** | | | |  |  |  |  |  |
|  | Albania | 5268 | 1550 | 1550 | 1550 | 541 | 149 | N/A |
|  | Colombia | 10686 | 6554 | 6554 | N/A | N/A | N/A | N/A |
|  | Jordan | 7832 | 5353 | 5353 | 5353 | 1945 | 968 | N/A |
|  | Maldives | 2824 | 1693 | 1693 | 1693 | 590 | 126 | N/A |
|  | South Africa | 2841 | 1977 | 1977 | 1977 | 670 | 350 | N/A |
| **Total** | | 247 232 | 354 605 | 354 605 | 348 051 | 125 286 | 80 572 | 188 046 |

**Notes:**

FP — family planning, ANC — antenatal care, PNC — postnatal care

All numbers are unweighted

^1^ Women age 15–49 years old who were sexually active, not currently pregnant, intended to space or limit pregnancy, and have not had sterilisation.

^2^ Women age 15–49 years old who had given live birth in three years preceding the survey

^3^ Children age 12–23 months

^4^ Children under-five years reported to have had diarrhoea in 2 weeks preceding the survey

^5^ Children under-five years in 14 malaria endemic countries (see Appendix 1)

**Appendix 3. Respondents’ background characteristics**

| **Variables** | **FP**^1^ | | **ANC/Delivery**^2^ | | **PNC**^2^ | | **Immunisation**^3^ | | **Diarrhoea treatment**^4^ | | **Insectiside-treated nets**^6^ | |
| --- | --- | --- | --- | --- | --- | --- | --- | --- | --- | --- | --- | --- |
| **Total** | **100%** | **247 232** | **100%** | **354 605** | **100%** | **348 051** | **100%** | **125 286** | **100%** | **80 572** | **100%** | **188 046** |
| **Women's age** |  |  |  |  |  |  |  |  |  |  |  |  |
| 15–25 years | 21% | 51 729 | 37% | 126 909 | 37% | 123 927 | N/A | N/A | N/A | N/A | N/A | N/A |
| 26–35 years | 39% | 95 460 | 49% | 174 267 | 49% | 171 554 | N/A | N/A | N/A | N/A | N/A | N/A |
| 33–49 years | 40% | 100 043 | 14% | 53 429 | 14% | 52 570 | N/A | N/A | N/A | N/A | N/A | N/A |
| **Women's age at delivery** |  |  |  |  |  |  |  |  |  |  |  |  |
| <20 years | N/A | N/A | 11% | 36 428 | 10% | 35 103 | 11% | 13 504 | 12% | 9 555 | 14% | 25 959 |
| 20–35 years | N/A | N/A | 80% | 283 734 | 80% | 279 050 | 80% | 100 586 | 79% | 63 551 | 74% | 138 373 |
| >35 years | N/A | N/A | 9% | 34 443 | 9% | 33 898 | 9% | 11 196 | 9% | 7 466 | 12% | 23 714 |
| **Sex of child** |  |  |  |  |  |  |  |  |  |  |  |  |
| Male | N/A | N/A | N/A | N/A | N/A | N/A | 51% | 64 413 | 53% | 42 660 | 51% | 94 705 |
| Female | N/A | N/A | N/A | N/A | N/A | N/A | 49% | 60 873 | 47% | 37 912 | 49% | 93 341 |
| **Parity** |  |  |  |  |  |  |  |  |  |  |  |  |
| 0–2 children | 46% | 108 946 | 56% | 192 777 | 55% | 188 065 | N/A | N/A | N/A | N/A | N/A | N/A |
| >2 children | 54% | 138 286 | 44% | 161 828 | 45% | 159 986 | N/A | N/A | N/A | N/A | N/A | N/A |
| **Education** |  |  |  |  |  |  |  |  |  |  |  |  |
| No education | 25% | 63 355 | 30% | 108 968 | 30% | 108 806 | 30% | 38 925 | 36% | 29 680 | 37% | 72 165 |
| Primary | 30% | 73 168 | 25% | 86 322 | 25% | 85 129 | 25% | 30 819 | 29% | 22 681 | 37% | 68 149 |
| Secondary | 34% | 84 450 | 36% | 127 372 | 36% | 123 988 | 36% | 44 748 | 30% | 23 730 | 22% | 40 842 |
| Higher/vocational | 11% | 26 259 | 10% | 31 943 | 9% | 30 128 | 9% | 10 794 | 6% | 4 481 | 4% | 6 890 |
| **Residency** |  |  |  |  |  |  |  |  |  |  |  |  |
| Urban | 40% | 95 981 | 32% | 106 380 | 31% | 101 825 | 31% | 36 525 | 28% | 22 577 | 31% | 56 937 |
| Rural | 60% | 151 251 | 68% | 248 225 | 69% | 246 226 | 69% | 88 761 | 72% | 57 995 | 69% | 131 109 |
| **Wealth index** |  |  |  |  |  |  |  |  |  |  |  |  |
| Poorest | 18% | 50 668 | 23% | 88 623 | 23% | 86 454 | 23% | 31 354 | 25% | 21 067 | 22% | 45 352 |
| Poor | 20% | 51 523 | 22% | 79 964 | 21% | 77 883 | 21% | 27 899 | 22% | 18 622 | 22% | 41 090 |
| Middle | 20% | 49 308 | 20% | 70 777 | 20% | 69 539 | 20% | 25 281 | 20% | 16 185 | 20% | 38 187 |
| Rich | 21% | 48 439 | 19% | 62 180 | 19% | 61 495 | 19% | 22 101 | 18% | 13 996 | 19% | 34 143 |
| Richest | 21% | 47 294 | 16% | 53 061 | 17% | 52 680 | 16% | 18 651 | 14% | 10 702 | 17% | 29 274 |
| **Income group** |  |  |  |  |  |  |  |  |  |  |  |  |
| Low | 37% | 90 443 | 31% | 105 350 | 31% | 105 350 | 31% | 37 466 | 43% | 33 704 | 54% | 99 457 |
| Lower-middle | 51% | 127 338 | 65% | 232 128 | 66% | 232 128 | 67% | 84 074 | 55% | 45 275 | 46% | 88 589 |
| Upper-middle | 12% | 29 451 | 5% | 17 127 | 3% | 10 573 | 3% | 3 746 | 2% | 1 593 | N/A | N/A |

Notes:

FP — family planning, ANC — antenatal care, PNC — postnatal care

All numbers are unweighted

^1^ Women age 15–49 years old who were sexually active, not currently pregnant, intended to space or limit pregnancy, and have not had sterilisation.

^2^ Women age 15–49 years old who had given live birth in three years preceding the survey

^3^ Children age 12–23 months

^4^ Children under-five years reported to have had diarrhoea in 2 weeks preceding the survey

^5^ All children under-five years reported to have had cough in last two weeks, short/rapid breaths, problem in the chest or blocked or running nose. in the two weeks before the survey

^6^ Children under-five years in 14 malaria endemic countries (see Appendix 1)

**Appendix 4. List of variables**

| **Variables** | **Type** | **Measurement** | **DHS measurement** |
| --- | --- | --- | --- |
| **OUTCOME VARIABLES** | | | |
| **1. Family Planning** | | | |
| **Target population:**  Married or sexually active non-married women who were not currently pregnant and intended to space or limit pregnancy and have not had sterilisation (including theirmale partner) | Binary | 1. No (never had sex, pregnant, declared infecund, intend to get pregnant, sterilized (including their partner)) 2. Yes (married/sexually active women who wanted to space or limit child birth, or still wanted to have more children but unsure the timing or had not decided)   Sexually active: married women or unmarried/ever-married women who had sexual intercourse in the last 4 weeks. | Q226: Are you currently pregnant?  q1 (household schedule): What is (NAME)'s current marital status?  Q714: When was the last time you had sexual intercourse?  Q804: Would you like to have (a/another) child, or would you prefer not to have any (more) children?  Q805: How long would you like b) to wait from now before the birth of (a/another) child? |
| **Service contact:**  Women who had contact with health service either by visiting a health facility or were visited by a health worker in the last 6-months (for any reason) | Binary | 1. No 2. Yes | Q327: In the last 6 months, were you visited by a fieldworker?  Q329: In the last months, have you visited a health facility?  In Bangladesh & India’s dataset, the period was in the last 3 months, instead of 6 months. |
| **Crude coverage:**  Women who were the target population of FP service and had contact with health service provider and were using any modern family planning method. | Binary | 1. Not using/traditional method 2. Modern method (pill, IUD, injections, diaphragm, condom, sterilisation (which would have been excluded through the ‘target population’ variable), Norplant (implant) and lactational amenorrhea) | Q307a: Are you currently using any contraceptive method?  Q306b: Which method are you using? |
| **Quality-adjusted coverage:**  Women who were the target population of FP service, had contact with health service provider, currently using any modern FP method and scored for all of the FP Methods Information Index | Binary | 1. No 2. Yes, received all method information index services   The methods information index is an indicator summarising whether service providers give adequate information to women when receiving FP services in order to assist with making informed choices.^[[3]](#footnote-3)^  The index consists of three questions:   1. Were you informed about other methods? 2. Were you informed about side effects? 3. Were you told what to do if you experienced side effects? | Method information index  Q322: were you told about other methods of family planning that you could use?  Q323: Were you ever told by a health or family planning worker about other methods of family planning that you could use?  Q318: At that time, were you told about side effects or problems you might have with the method?  Q320: Were you ever told by a health or family planning worker about side effects or problems you might have with the method?  Q321: Were you told what to do if you experienced side effects or problems?  Methods information index is not available in Bangladesh. |
| **2. Antenatal care (ANC)** | | | |
| **Target population:**  Women who had recently given a livebirth/s within the last 3-years of each survey wave | Binary | 1. No 2. Yes | Q402: Number of births in the last five years |
| **Service contact:**  Women with a recent live birth within 3-years prior to each survey wave and received any number of ANC service from skilled providers. |  | 1. No 2. Yes, received any ANC from skilled providers   Skilled health professionals for ANC service include: general practitioner, obstetrician, nurse, midwife and village midwife. | Q408: Did you see anyone for antenatal care for this pregnancy?  Q409: Whom did you see? |
| **Crude coverage:**  Women with a recent live birth within 3-years prior to each survey wave and received at least 4 times ANC services from skilled providers | Binary | 1. No, < 4 visit(s) 2. Yes, ≥ 4 visits | Q412: How many times did you receive antenatal care during this pregnancy? |
| **Quality-adjusted coverage:**  Women with a recent live birth within 3-years prior to each survey wave and received at least 4 times ANC services and key components of ANC from a health provider, which include: weight and height measured, blood pressure, urine sample and blood sample taken, given/bought iron tablets/syrup | Binary | 1. No 2. Yes, received all the four services | Q413: As part of your antenatal care during this pregnancy, were any of the following done at least once?  a) Was your blood pressure measured? b) Did you give a blood sample? c) Did you give a urine sample?  q420: During this pregnancy, were you given or did you buy any iron tablets or iron syrup?  Notes:   - we only measured services that were consistently measured in almost all countries. - iron-tablets/syrup supplementation **are not-available** in **Bangladesh and Colombia** |
| **User-adherence-adjusted coverage:**  Women with a recent live birth within 3-years prior to each survey wave and received at least 4 times ANC services and key components of ANC from a health provider, which include: blood pressure, urine sample and blood sample taken, given/bought iron tablets/syrup and adhered to consuming iron supplementation for at least 90 days during the pregnancy | Binary | 1. No 2. Yes, iron supplementation for at least 90 days during the pregnancy | Q421: During the whole pregnancy, for how many days did you take the tables or syrup? |
| **3. Delivery care** | | | |
| **Target population:**  Women who had recently given a livebirth/s within the last 3-years of each survey wave | Binary | 1. No 2. Yes | Q402: Number of births in the last five years |
| **Service contact:**  Delivery attended by a skilled health provider | Binary | 1. No 2. Yes, skilled birth attendant   Skilled birth attendant includes general practitioner, obstetrician, nurse, midwife and village midwife | Q429: Who assisted with the delivery of (child's name)? |
| **Crude coverage:**  Skilled birth attendance at a health facility | Binary | 1. No 2. Yes   Health facility includes hospital, clinic, health post, maternity home, OB-GYN clinic, Midwife/nurse clinic | Q430: Where did you give birth to (child's name)? |
| **4. Postnatal care (PNC)** | | | |
| **Target population:**  Women who had recently given a livebirth/s within the last 3-years of each survey wave | Binary | 1. No 2. Yes | Q402: Number of births in the last five years |
| **Service contact:**  Women with a recent live birth within 3-years prior to each survey wave received postnatal care for their newborn from skilled providers at any period following birth. | Binary | 1. No 2. Yes, with skilled providers   All deliveries attended by SBA was included as having PNC service contact. | Q429: Who assisted with the delivery of (child's name)?  Q438: Did anyone check on  (NAME)’s health while you were still in  the facility?  Q440: Who checked on (NAME)’s health at that  time? |
| **Crude coverage:**  Women with a recent live birth within 3-years prior to each survey wave received postnatal care for their newborn in the first 24 hours after birth from skilled providers | Binary | 1. No 2. Yes, in the first 24 hours | Q439: How long after delivery was (NAME)’s  health first checked? |
| **Quality-adjusted coverage:**  Women with a recent live birth within 3-years prior to each survey wave received postnatal care for their newborn in the first 24 hours after birth from skilled providers, weighed, and received BCG vaccination before 1 months. | Binary | 1. No 2. Yes, weighed and received BCG vaccination (<1 mos) ^[[4]](#footnote-4)^ | Q427: Was (NAME) weighed at birth?  Q512A: Has (NAME) ever received a BCG vaccination against tuberculosis, that is, an injection in the arm or shoulder that usually causes a scar?  Q508B: Days, month, year |
| **5. Child Immunisation** | | | |
| **Target population:**  All children aged 12-24 women who were alive during the survey | Binary | 1. No 2. Yes | QINTD: Date of interview  Q215D: On what day,  month, and year was (NAME) born? (year)  Age of the child = QINTCD – Q215D  Q216: is (NAME) still alive? |
| **Service contact:**  Children aged 12-24 women who were alive during the survey who received DPT-1 vaccination. | Binary | 1. No 2. Yes, received DPT vaccination.   A child is considered to have received DPT Immunisation if either: (1) the vaccination date was reported on the Immunisation card, (2) the mother reported that the vaccination was given or (3) the vaccination was marked on the Immunisation card | Q517b: Has (NAME) ever received a DPT vaccination, that is, an injection to prevent diphtheria, pertussis and tetanus, given in the thigh sometimes at the same time as polio drops? |
| **Crude coverage:**  Children aged 12-24 women who were alive during the survey who received DPT-1, DPT-2 and DPT-3 vaccination | Binary | 1. Did not received DPT-2 or DPT-3 2. Received all the three DPT vaccination (DPT-1, DPT-2, and DPT-3) | Q517aa: How many times did (NAME) receive the DPT vaccine? |
| **Quality-adjusted coverage:**  Children aged 12-24 women who were alive during the survey who received DPT-1, DPT-2, DPT-3 and measles vaccination | Binary | 1. No 2. Yes, all DPT and received measles vaccination | Q523a: Has (NAME) ever received a measles vaccination, that is, an injection in the arm to prevent measles? |
| **User-adherence-adjusted coverage:**  Children aged 12-24 women who were alive during the survey who received DPT-1, DPT-2, DPT-3 and measles vaccination according to the WHO Immunisation schedule | Binary | 1. No 2. Yes, adhered to the schedule | Q508B: Days, month, year  According to WHO child Immunisation guideline^2^, schedule for:  1) DPT-1: at 2 months 2) DPT-2: at 4 months 3) DPT-3: at 6 months 4) Measles: at 6-12 months |
| **6. Diarrhoea treatment** | | | |
| **Target population:**  All children under 5 years reported to have had diarrhoea in the 2 weeks before the survey | Binary | 1. No 2. Yes | QINTD: Date of interview  Q215D: On what day,  month, and year was (NAME) born? (year)  Age of the child = QINTCD – Q215D  Q608: Has (NAME) had Diarrhoea in the last 2 weeks? |
| **Service contact:**  All children under 5 years reported to have had symptoms  in the 2 weeks before the survey and seek treatment from health facility or provider | Binary | 1. No 2. Yes, from health facility/provider   Health facility or providers include: hospital, health centre, mobile clinic, fieldworker. | Q611: Did you seek advice or treatment for the  Diarrhoea from any source? |
| **Crude contact:**  All children under 5 years reported to have had symptoms  in the 2 weeks before the survey and seek treatment and received oral rehydration therapy (ORT)/increased fluids from health facility or provider | Binary | 1. No 2. Yes, received ORS / a pre-packaged / ORS/homemade fluid   Oral rehydration therapy includes children who treated by a fluid made from a special packet of oral rehydration salts (ORS), received government-recommended homemade fluids (RHF), or received increased fluids. | Q617: Was (NAME) given any of the following at any time since (NAME) started having the Diarrhoea:  a) A fluid made from a special packet called [LOCAL NAME FOR ORS  PACKET]?  b) A pre-packaged ORS liquid?  c) A government-recommended  homemade fluid? |
| **Quality contact:**  All children under 5 years reported to have had symptoms  in the 2 weeks before the survey and seek treatment and received the ORS | Binary | 1. No 2. Yes, received ORS/pre-packed ORS^[[5]](#footnote-5)^   We did not include zinc as the treatment of diarrhoea since it is not consistently available | Q617: Was (NAME) given any of the following at any time since (NAME) started having the Diarrhoea:  a) A fluid made from a special packet called [LOCAL NAME FOR ORS  PACKET]?  b) A pre-packaged ORS liquid? |
| **User-adherence-adjusted coverage:**  All children under 5 years reported to have had symptoms  in the 2 weeks before the survey and seek treatment and received the ORS and continued feeding | Binary | 1. No 2. Yes, received ORS and continued feeding^3^   Continued feeding includes children who were given more, the same as usual during the Diarrhoea episode. | Q617: When (NAME) had Diarrhoea, was (NAME) given less than usual to eat, about the same amount, more than usual, or nothing to eat? |
| **7. Use of insecticide treated nets** | | | |
| **Target population:**  All children under-5 years in Malaria high risk area | Binary | 1. No 2. Yes, aged <5 years and lived in Malaria high risk area (see Appendix 1)^[[6]](#footnote-6)^ | QINTD: Date of interview  Q215D: On what day,  month, and year was (NAME) born? (year)  Age of the child = QINTCD – Q215D |
| **Service contact:**  All children under-5 years in Malaria high risk area that owned an ITN | Binary | 1. No 2. Yes | Household questionnaire  Q127: Does your household have any mosquito nets? |
| **Crude coverage:**  All children under-5 years in Malaria high risk area that owned an ITN and slept under an insecticide-treated net in the last night before the survey | Binary | 1. No 2. Yes, children slept under mosquito net last night | Q137: Who slept under this mosquito net last night? |
| **INDEPENDENT VARIABLES** | | | |
| Wealth index | Categorical ordinal | 1. Poorest / least wealthy 2. Poor 3. Middle 4. Rich 5. Richest / wealthiest | The wealth index is a composite measure of a household's cumulative living standard.  The wealth index is calculated using easy-to-collect data on a household’s ownership of selected assets, such as televisions and bicycles; materials used for housing construction; and  types of water access and sanitation facilities. The wealth index is presented in the DHS Final Reports and survey datasets as a background characteristic. |
| Education | Categorical ordinal | 1. Primary/lower 2. Secondary 3. Tertiary/higher | Q107: Have you ever attended school?  Q108: What is the highest level of school you attended: primary, junior high, senior high, academy or university?  Q109: What is the highest (grade/year) you completed at that level? |
| **COVARIATES** | | | |
| Women’s age | Categorical ordinal | 1. 15-25 years 2. 26-35 years 3. 36-42 years 4. 42-49 years | Q105: In what month and year were you born? |
| Women’s age at delivery | Categorical ordinal | 1. <20 years 2. 20–35 years 3. >35 years   Age at delivery = (CMC of birth of child - CMC of birth of mother) / 12 months | Q215: In what month  and year was (NAME) born? (recorded in Century Month Code(CMC))  Q105: In what month and year were you born? (recorded in CMC) |
| Parity | Categorical ordinal | 1. 0–2 children 2. > 2 children | BORD: Birth order number |
| Sex of child | Binary | 1. Male 2. Female | Q213: Is (NAME) a boy or a girl? |
| Residency | Binary | 1. Rural 2. Urban | Q5: Urban/rural (identified by interviewers) |
| Covariates used for specific outcomes:   1. Family planning: Women’s age, parity, and residency 2. ANC, delivery care, and PNC: Age at delivery, parity, and residency 3. Immunisation and insecticide treated nets: women’s age at delivery, sex of child, and residency | | | |

**Appendix 5. Effective coverage indicators in the previous studies^7,9^**

| **Intervention** | **Target Population** | **Service contact** | **Crude coverage** | **Quality-adjusted coverage** | **User-adherence adjusted coverage** |
| --- | --- | --- | --- | --- | --- |
| **Family planning** | **The effective coverage indicators by Amouzou, et al.^[[7]](#footnote-7)^** | | | | |
|  | Sexually active  women who do  not intend to  become pregnant | Woman visits  health facility  (for any reason) | Receives FP methods | Multiple methods  choice; standards  followed | Use modern methods  according to protocol |
|  | **This study** | | | | |
|  | Women aged 15–49 years old who were sexually active, not currently pregnant, intended to space or limit pregnancy, and have not had sterilisation. | Women who visited  the health facility in last 12-months (for any reason) or,  women who had been visited by FP worker in the last 12 months | Use a modern family planning method | Receive information about:   - side-effect of the current methods - how to deal with the side effects - other FP methods   Notes:  The methods information index is an indicator summarising whether service providers give adequate information to women when receiving FP services in order to assist with making informed choices.^[[8]](#footnote-8)^ | N/A  Notes:  Cannot be measured. |
| **Antenatal visit** | **The effective coverage indicators by Amouzou, et al.^7^** | | | | |
|  | Women who are  pregnant | Visits ANC clinic | Receives ANC  interventions | All relevant  interventions and  according to standard | Use of selected  interventions at home |
|  | **This study** | | | | |
|  | Women age 15–49 years old who had given live birth in three years preceding the survey | Use any number of ANC service from skilled providers.  Notes:  We assume that those who received first ANC had access to visit ANC clinic | Use at least 4 times ANC service | Use at least 4 times and receive key components of ANC  Components of ANC:   - Blood pressure taken - Urine sample taken - Blood sample taken - Given/bough iron tablets/syrup   Notes:  We only selected four components of ANC that are consistently measured in all countries. These components are used as the proxy of quality of ANC.^[[9]](#footnote-9)^ | Receive quality-adjusted ANC service AND adhered to consuming iron tablets for ≥ 90 days during pregnancy |
| **Delivery care** | **The effective coverage indicators by Amouzou, et al.^7^** | | | | |
|  | Women who are  delivering | Deliver in a health facility | Received delivery care | Receives all required  delivery interventions  according to  standards | N/A |
|  | **This study** | | | | |
|  | Women age 15–49 years old who had given live birth in three years preceding the survey | Skilled birth attendance (SBA)  Notes:  We assume that those who delivered under supervision of skilled birth attendance had access to the delivery service. | Skilled birth attendance at a health facility | N/A | N/A |
| **Postnatal care** | **The effective coverage indicators by Amouzou, et al. ^7^** | | | | |
|  | Women who  have delivered;  newborns | Visits PNC clinic | Receives PNC  interventions | Receives PNC  interventions  according to standard | N/A |
|  | **This study** | | | | |
|  | Women age 15–49 years old who had recently given live birth in three years preceding the survey | Newborn receive postnatal care from a skilled provider.  Notes:  All births attended by skilled providers were included as having PNC service contact. Women who received SBA are assumed to have access to PNC service. | Receive postnatal care in the first 24 hours after birth or delivery attended by SBA. | Receive postnatal care in the first 24 hours after birth from health providers, weighed, and received BCG vaccination before 1 month.  Notes:  We only selected components that are consistently measured in all countries as a proxy of the quality of PNC service. | N/A |
| **Immunisation** | **The effective coverage indicators by Amouzou, et al. ^7^** | | | | |
|  | infants at  different ages | Infant visits  health facility | Receives vaccination | Receives vaccination  according to  standards | Timely vaccination  according to age and  standard |
|  | **This study** | | | | |
|  | Children age 12–23 months  Notes:  We followed DHS sample and definition in measuring the coverage of Immunisation. | Received DPT-1  Notes:  We assume that those who had received DPT-1 had awareness/access to Immunisation service. | Receive three doses of DPT-containing vaccines | Receive three doses of DPT-containing vaccines and one dose of measles vaccine.  Notes:  We only included DPT and measles in our analysis to maintain consistency of doses and Immunisation schedule across countries. | Receive all timely vaccination:   - DPT: 2,4,6 months - Measles: $\leq$ 12 months. |
| **Management of diarrhoea** | **The effective coverage indicators by Amouzou, et al. ^7^** | | | | |
|  | Children with  diarrhoea | Taken to health  facility | ORS received (and  other treatment/  advice) | ORS received  according to  standards | Use of intervention at  home |
|  | **This study** | | | | |
|  | Children under-five years reported having had diarrhoea in 2 weeks preceding the survey  Notes:  We followed DHS sample and definition in measuring the prevalence of diarrhoea. | Seek treatment from a health facility or provider | Receive oral rehydration therapy (ORT) or increased fluids.  Notes:  Oral rehydration therapy includes children who treated by a fluid made from a special packet of oral rehydration salts (ORS), received government-recommended homemade fluids (RHF), or received increased fluids. | Receive the oral rehydration salt mixture  Notes:  We only included children who received ORS/prepacked ORS for this stage. We did not include zinc as the treatment of diarrhoea since this variable is not consistently available. | Receive ORS and continued feeding.  Notes:  Continue feeding = feeding children with Diarrhoea normally and to increase the amount of fluids given. |
| **Use of insecticide-treated nets** | **The effective coverage indicators by Nguhiu, et al.^[[10]](#footnote-10)^** | | | | |
|  | All children and pregnant  women | Proportion of children and pregnant  women living in household that  owned an ITN | Proportion of  children and pregnant women who actually slept under an insecticide treated net in the preceding night | N/A | N/A |
|  | **This study** | | | | |
|  | Children under-five years in 14 malaria-endemic countries (see Appendix 1) | Owned an ITN | Slept under an insecticide-treated net in the preceding night | N/A | N/A |

**Appendix 6. List of skilled providers by countries**

| **Countries** | **DHS Year** | **Skilled provider - ANC** | **Skilled provider - Delivery** | **Skilled provider - Postnatal** |
| --- | --- | --- | --- | --- |
| **Afghanistan** | 2015 | Doctor | Doctor | Doctor |
|  |  | Nurse/midwife | Nurse/midwife | Nurse/midwife |
|  |  | Auxiliary nurse/midwife | Auxiliary nurse/midwife | Auxiliary nurse/midwife |
| **Albania** | 2017 | Obstetrician/gynecologist | Obstetrician/gynecologist | Obstetrician/gynecologist |
|  |  | Family doctor | Family doctor | Family doctor |
|  |  | Nurse/midwife | Nurse/midwife | Nurse/midwife |
| **Angola** | 2015 | Doctor/medico | Doctor/medico | Doctor/medico |
|  |  | Nurse/enfermeira | Nurse/enfermeira | Nurse/enfermeira |
|  |  | Birth attendance/parteira | Birth attendance/parteira | Birth attendance/parteira |
| **Armenia** | 2016 | Doctor | Doctor | Doctor |
|  |  | Nurse/midwife | Nurse/midwife | Nurse/midwife |
|  |  | Feldsher | Feldsher | Auxiliary midwife |
| **Bangladesh** | 2014 | Qualified doctor | Qualified doctor | Qualified doctor |
|  |  | Nurse/midwife/paramedic | Nurse/midwife/paramedic | Nurse/midwife/paramedic |
|  |  | FMW | FMW | FMW |
|  |  | CSBA | CSBA | CSBA |
|  |  | SACMO | SACMO | SACMO |
| **Benin** | 2017 | Doctor/medecins | Doctor/medecins | Doctor/medecins |
|  |  | Nurse/infirmiers | Nurse/informieres | Nurse/informieres |
|  |  | Midwife/sages-femmes | Midwife/sages-femmes | Midwife/sages-femmes |
| **Burundi** | 2016 | Physicians/medecins | Physicians | Physicians |
|  |  | Nurse/infirmiers | Nurse | Nurse |
|  |  | Midwife/sages-femmes | Midwife | Midwife |
| **Cambodia** | 2014 | Doctor | Doctor | Doctor |
|  |  | Nurse | Nurse | Nurse/midwife |
|  |  | Midwife | Midwife | Auxiliary midwife |
| **Cameroon** | 2018 | Physicians/medecins | Physicians/medecins | Physicians/medecins |
|  |  | Nurse/infirmiers | Nurse/infirmiers | Nurse/infirmiers |
|  |  | Midwife/sages-femmes | Midwife/sages-femmes | Midwife/sages-femmes |
|  |  | aides-soignants | aides-soignants | aides-soignants |
| **Chad** | 2014 | Doctor | Doctor | Doctor |
|  |  | Nurse/midwife | Nurse/midwife | Nurse/midwife |
|  |  | Matron | Matron | Auxiliary midwife/hospital health center worker |
|  |  | Hospital/health center officer | Hospital/health center officer | Trained birth attendant |
| **Colombia** | 2015 | Doctor/medico | Doctor/medico | Doctor/medico |
|  |  | Nurse/enfermeira | Nurse/enfermeira | Nurse/enfermeira |
| **Ethiopia** | 2016 | Doctor | Doctor | Doctor |
|  |  | Nurse | Nurse | Nurse |
|  |  | Midwife | Midwife | Midwife |
|  |  | Health officers | Health officers | Health officers |
|  |  | Health extension worker | Health extension worker | Health extension worker |
| **Ghana** | 2014 | Doctor | Doctor | Doctor |
|  |  | Nurse/midwife | Nurse/midwife | Nurse/midwife |
|  |  | Community health officer | Community health officer | Community health officer/nurse |
| **Guatemala** | 2015 | Doctor/medico | Doctor/medico | Doctor/medico |
|  |  | Ambulatory doctor | Ambulatory doctor | Ambulatory doctor |
|  |  | Nurse/enfermera | Nurse/enfermera | Nurse/enfermera |
|  |  | *midwife/comadrona capacitada --> unskilled |  |  |
| **Guinea** | 2018 | Doctor/medecins | Doctor/medecins | Doctor/medecins |
|  |  | Nurse/Midwife, infirmiers/sages-femmes | Nurse/Midwife, infirmiers/sages-femmes | Nurse/Midwife, infirmiers/sages-femmes |
|  |  | Technical health officer/agents techniques de sante | Technical health officer/agents techniques de sante | Technical health officer/agents techniques de sante |
| **Haiti** | 2016 | Physicians / medecins | Physicians | Physicians |
|  |  | Nurse / infirmieres | Nurse | Nurse |
|  |  | Midwifery nurse / sage-femmes | Midwifery nurse | Midwifery nurse |
|  |  | Nurse assistant/infirmières/sages-femmes et auxiliaires | Nurse assistant/infirmières/sages-femmes et auxiliaires --> non skilled for delivery | Auxiliary midwife |
| **India** | 2015 | Doctor | Doctor | Doctor |
|  |  | Anm/nurse/midwife/LHV | Anm/nurse/midwife/LHV | Anm/nurse/midwife/LHV |
| **Indonesia** | 2017 | Doctor | Doctor | Doctor |
|  |  | Obstetrician | Obstetrician | Obstetrician |
|  |  | Nurse | Nurse | Nurse |
|  |  | Midwife | Midwife | Midwife |
|  |  | Village midwife | Village midwife | Village midwife |
| **Jordan** | 2017 | Doctor | Doctor | Doctor |
|  |  | Nurse/Midwife | Nurse/Midwife | Nurse/midwife |
| **Kenya** | 2014 | Doctor | Doctor | Doctor |
|  |  | Nurse/midwife | Nurse/midwife | Nurse/midwife |
| **Lesotho** | 2014 | Doctor | Doctor | Doctor |
|  |  | Nurse/midwife | Nurse/midwife | Nurse/midwife |
| **Malawi** | 2015 | Doctor/clincal officer/medical assistant | Doctor/clincal officer/medical assistant | Doctor/clincal officer/medical assistant |
|  |  | Nurse/midwife | Nurse/midwife | Nurse/midwife |
| **Maldives** | 2016 | Gynecologist | Gynecologist | Doctor |
|  |  | Doctor | Doctor | Nurse/midwife |
|  |  | Nurse/midwife | Nurse/midwife |  |
| **Mali** | 2018 | Doctor | Doctor | Doctor |
|  |  | Nurse/midwife | Nurse/midwife | Nurse/midwife |
|  |  | Matron | Matron | Auxiliary midwife |
| **Myanmar** | 2016 | Doctor | Doctor | Doctor |
|  |  | Nurse/midwife/LHV | Nurse/midwife/LHV | Nurse/midwife/LHV |
| **Nepal** | 2016 | Doctor | Doctor | Doctor |
|  |  | Nurse/midwife | Nurse/midwife | Nurse/midwife |
|  |  |  |  | Auxiliary midwife |
| **Nigeria** | 2018 | Doctor | Doctor | Doctor |
|  |  | Nurse/Midwife | Nurse/Midwife | Nurse/midwife |
|  |  | Auxiliary midwife | Auxiliary midwife | Auxiliary midwife |
| **Pakistan** | 2017 | Doctor | Doctor | Doctor |
|  |  | Nurse/midwife/LHV | Nurse | Nurse |
|  |  |  | Midwife | Midwife |
|  |  |  | LHV | LHV |
|  |  |  | Community midwife | Community midwife |
| **Papua New Guinea** | 2017 | Doctor | Doctor | Doctor |
|  |  | Midwife | Midwife | Midwife |
|  |  | Nurse | Nurse | Nurse |
|  |  | Trained village health volunteer | Trained village health volunteer | Trained village health volunteer |
| **Philippines** | 2017 | Doctor | Doctor | Doctor |
|  |  | Nurse | Nurse | Nurse/midwife |
|  |  | Midwife | Midwife | Auxiliary midwife |
| **Rwanda** | 2015 | Doctor | Doctor | Doctor |
|  |  | Nurse/medical assistant | Nurse/medical assistant | Nurse/medical assistant |
|  |  | Midwife | Midwife | Midwife |
| **Senegal** | 2017 | Doctor | Doctor | Doctor |
|  |  | Nurse | Nurse | Nurse |
|  |  | Midwife | Midwife | Midwife |
| **South Africa** | 2016 | Doctor/gynecologist | Doctor/gynecologist | Doctor/gynecologist |
|  |  | Nurse/midwife | Nurse/midwife | Nurse/midwife |
| **Tajikistan** | 2017 | Doctor | Doctor | Family doctor |
|  |  | Family doctor | Family doctor | Obgyn |
|  |  | Obgyn | Obgyn | Other doctor |
|  |  | Other doctor | Other doctor | Nurse |
|  |  | Nurse | Nurse | Midwife |
|  |  | Midwife | Midwife |  |
| **Tanzania** | 2015 | Doctor | Doctor/AMO | Doctor/AMO |
|  |  | Clinical oficer | Clinical officer | Clinical officer |
|  |  | Assistant clinical officer | Assistant clinical officer | Assistant clinical officer |
|  |  | Nurse/midwife | Nurse/midwife | Nurse/midwife |
|  |  | Assistant nurse | Assistant nurse | Assistant nurse |
|  |  | MCH Aide | MCH Aide | MCH Aide |
| **Timor Leste** | 2016 | Doctor | Doctor | Doctor |
|  |  | Nurse/midwife | Nurse/midwife | Nurse/midwife |
|  |  | Assistant nurse | Assistant nurse | Assistant nurse |
| **Uganda** | 2016 | Doctor | Doctor | Doctor |
|  |  | Nurse/midwife | Nurse/midwife | Nurse/midwife |
|  |  | Medical assistant/clinical officer | Medical assistant/clinical offcier | Medical assistant/clinical offcier |
| **Zambia** | 2018 | Doctor | Doctor | Doctor |
|  |  | Nurse/midwife | Nurse/midwife | Nurse/midwife |
|  |  | Medical assistant/clinical officer | Medical assistant/clinical officer | Medical assistant/clinical officer |
| **Zimbabwe** | 2015 | Doctor | Doctor | Doctor |
|  |  | Nurse | Nurse | Nurse |
|  |  | Midwife | Midwife | Midwife |

**Appendix 7. The measurement of socioeconomic inequalities**

**Simple measures of inequality**

- Difference (diff): unweighted measure of absolute inequality between two groups.

$$Diff=y_{high}- y_{low}$$

- Ratio (R): unweighted measure of relative inequality between two groups

$$R=y_{high} / y_{low}$$

$y_{high}$ refers to the most-advantaged subgroup (wealthiest or most educated) and $y_{low}$ refers to the most-disadvantaged subgroup (least wealthy or least educated)

**Complex measures of inequality**

- Slope index of inequality (SII): weighted measure of absolute difference in estimated values of an indicator between the most-advantaged and most-advantaged, while taking into account the distribution of entire socioeconomic groups. Positive values (SII>0) indicate higher level of inequality among most-advantaged subgroups.
- Relative index of inequality (RII): weighted measure of relative difference in estimated values of an indicator between the most-advantaged and most-disadvantaged, while taking into account the distribution of entire socioeconomic groups. RII estimates higher than one (RII>1) indicate higher level of inequality favouring the advantaged groups.

To estimate the SII and RII, we first rank individuals from highest (rank 1) to lowest (rank 0) socioeconomic status (i.e., wealth index and education level). The population of each socioeconomic category is assigned a fractional rank, called r score or ridit score, based on the 1 midpoint of its range in cumulative distribution of the population. For example, if respondents from the wealthiest groups (Q5) comprise 15% of the population, 0.075 (0.15/2) is assigned to respondents in this category. Furthermore, if the respondents from the wealthier group (Q4) makes up 20% of the population, the corresponding rank score is 0.25 (0.15 + [0.20 / 2]). We calculated the r score using *wridit* command in Stata. We then used generalised linear models (GLM) with a Poisson distribution and log-link function for RII, and identity link function for SII, to generate inequality measures that has been suggested by previous studies (Eq. 1).

$g\left( Y \right)= \beta_{0}+\beta_{1}rscore+\Sigma_{k}\beta_{k}X_{k}+ \varepsilon$ (Eq. 1)

where Y = 1 if outcome is present and Y = 0 if absent, g(Y) = Y is the identity-link function generating the SII, while g(Y)=log(Y) is log-link function generating the RII. β1 and βk refer to the regression coefficients and $rscore$ refers to the rank score for each respondent. $X_{k}$a set of k covariates in the model (see S5 Table for the list of covariates applied).

- Concentration index (CI): weighted measure of inequality that shows the gradient across multiple subgroups with natural ordering, on a relative scale. The concentration index can be expressed using Eq. 2 and Eq.3:

$CI(y)= \frac{2*cov ( y_{i}, R_{i})}{\mu}$ (Eq. 2)

where $y_{i}$ is the outcome variable in which inequality is measured, $\mu$ is the weigted mean of health variable, cov denotes the weighted covariance and $R_{i}$ is the relative fractional rank of the i^th^ individual in socioeconomic distribution (i.e., wealth index and education). Taking into account the binary nature of the outcome variables, Erreygers’ normalized concentration index was selected over the conventional CI.

$ECI(y)= \frac{4 \mu}{b_{n}-a_{n}}CI(y)$ (Eq. 3)

Where CI(y) is the standard concentration index, $\mu$ is the mean of the health variable, and $b_{n}$ and $a_{n}$ are the upper and lower bounds of health service coverage, respectively. If the concentration index is significantly greater than zero (CI>0), the outcome is concentrated in most advantaged individuals. Concentration index lower than zero (CI<0) indicate the outcome is concentrated in most disadvantaged individuals

**Appendix 8. Coverage by income groups**

| **Variables** | | **Service contact** | | **Crude coverage** | | **Quality-adjusted coverage** | | **User-adherence coverage** | | **Target population** |
| --- | --- | --- | --- | --- | --- | --- | --- | --- | --- | --- |
|  |  | **%** | **(95% CI)** | **%** | **(95% CI)** | **%** | **(95% CI)** | **%** | **(95% CI)** | **N** |
| **Family planning** | |  |  |  |  |  |  |  |  |  |
|  | **Overall: 39 countries** | **58·0** | **(57·4–58·7)** | **32·4** | **(31·9–32·9)** | **9·3** | **(9·0–9·6)** |  |  | **247 232** |
|  | Low income | 66·2 | (65·3–67·2) | 33·9 | (33·0–34·8) | 14·6 | (14·0–15·1) |  |  | 90 443 |
|  | Lower-middle income | 54·8 | (53·9–55·6) | 30·5 | (30·0–31·1) | 7·11 | (6·8–7·4) |  |  | 127 338 |
|  | Upper-middle income | 68·3 | (67·0–69·6) | 49 | (47·5–50·4) | 17 | (15·9–18·1) |  |  | 29 451 |
| **Antenatal care** | |  |  |  |  |  |  |  |  |  |
|  | **Overall: 39 countries** | **80·7** | **(81·7–81·2)** | **53·4** | **(52·8–54·0)** | **37·1** | **(36·6–37·7)** | **24·4** | **(23·9–24·8)** | **354 605** |
|  | Low income | 80·5 | (80·2–81·8) | 44·1 | (43·0–45·2) | 23·7 | (22·8–24·5) | 12·1 | (11·5–12·6) | 105 350 |
|  | Lower-middle income | 80·2 | (79·7–80·8) | 54·6 | (53·9–55·3) | 39·0 | (38·4–39·6) | 26·5 | (25·3–26·4) | 232 128 |
|  | Upper-middle income | 93·5 | (92·3–94·6) | 82·5 | (80·9–84·1) | 75·0 | (73·3–76·7) | 54·3 | (51·6–56·9) | 17 127 |
| **Delivery care** | |  |  |  |  |  |  |  |  |  |
|  | **Overall: 39 countries** | **71·6** | **(70·9–72·3)** | **67·3** | **(66·6–68·0)** |  |  |  |  | **354 605** |
|  | Low income | 61·3 | (59·6–62·9) | 58·9 | (57·2–60·5) |  |  |  |  | 105 350 |
|  | Lower-middle income | 73·2 | (72·4–73·9) | 68·2 | (67·5–69·0) |  |  |  |  | 232 128 |
|  | Upper-middle income | 97·0 | (96·4–97·6) | 96·1 | (95·4–96·7) |  |  |  |  | 17 127 |
| **Postnatal care** | |  |  |  |  |  |  |  |  |  |
|  | **Overall: 38 countries** | **75·1** | **(74·4–75·7)** | **38·2** | **(37·5–38·8)** | **11·1** | **(10·8–11·4)** |  |  | **348 051** |
|  | Low income | 67·1 | (65·4–68·8) | 36·2 | (35·0–37·4) | 14·6 | (13·9–15·2) |  |  | 105 350 |
|  | Lower-middle income | 76·4 | (75·7–77·1) | 37·3 | (36·6–38·0) | 9·5 | (9·2–9·8) |  |  | 232 128 |
|  | Upper-middle income | 99·3 | (99·0–99·7) | 84·8 | (83·0–86·7) | 35·0 | (32·2–37·9) |  |  | 10 573 |
| **Vaccination** | |  |  |  |  |  |  |  |  |  |
|  | **Overall: 38 countries** | **85·8** | **(85·3–86·3)** | **73·8** | **(73·2–74·5)** | **66·9** | **(66·1–67·6)** | **30·8** | **(30·2–31·5)** | **125 286** |
|  | Low income | 84·4 | (83·2–85·5) | 71·2 | (69·6–72·7) | 62·0 | (60·5–63·6) | 33·0 | (31·7–34·3) | 37 466 |
|  | Lower-middle income | 86·0 | (85·4–86·6) | 74·6 | (73·9–75·4) | 68·1 | (67·3–68·9) | 30·2 | (29·5–31·0) | 84 074 |
|  | Upper-middle income | 90·3 | (87·8–92·7) | 70·2 | (66·2–74·2) | 65·7 | (61·8–69·7) | 32·3 | (28·4–36·2) | 3 746 |
| **Diarrhoea treatment** | |  |  |  |  |  |  |  |  |  |
|  | **Overall: 38 countries** | **67·3** | **(66·5–68·0)** | **45·8** | **(45·0–46·7)** | **38·4** | **(37·6–39·1)** | **14·5** | **(13·9–15·0)** | **80 572** |
|  | Low income | 58·1 | (56·6–59·5) | 41·4 | (40·2–42·6) | 34·2 | (33·0–35·3) | 13·0 | (12·3–13·7) | 33 704 |
|  | Lower-middle income | 70·8 | (69·9–71·7) | 47·2 | (46·2–48·3) | 39·8 | (38·9–40·8) | 15·0 | (14·3–15·6) | 45 275 |
|  | Upper-middle income | 61·4 | (56·1–66·7) | 54·8 | (49·5–60·1) | 40·6 | (34·9–46·4) | 16·6 | (12·6–20·5) | 1 593 |
| **Use of insecticide-treated nets** | | |  |  |  |  |  |  |  |  |
|  | **Overall: 14 countries** | **74·4** | **(73·7–75·2)** | **61·4** | **(60·6–62·3)** |  |  |  |  | 188 046 |
|  | Low income | 80·7 | (79·8–81·6) | 68·1 | (67·1–69·1) |  |  |  |  | 99 457 |
|  | Lower-middle income | 70·9 | (69·9–71·9) | 57·7 | (56·6–58·8) |  |  |  |  | 88 589 |

**Appendix 9. Coverage of RMNCH services by country**

**Appendix 10-1. Simple measures of socioeconomic inequality based on wealth index**

***(Upper-middle-income countries)***

ANC — antenatal care, PNC — postnatal care, CI — confidence interval

Difference (Diff) = % coverage in most advantaged (wealthiest/tertiary education) – % coverage in most disadvantaged (least wealthy/primary or lower education).

Diff > 0 indicates most advantaged group reported higher coverage of RMNCH services.

Ratio = % coverage in most advantaged / % coverage in most disadvantaged. Ratio > 1 indicates most advantaged group were more likely to receive the RMNCH services.

Dark green indicates smaller inequality, red indicates higher inequality, grey indicates data not available/applicable.

**Appendix 10-2. Simple measures of socioeconomic inequality based on wealth index, by countries**

***(Lower-middle-income countries)***

Difference (Diff) = % coverage in most advantaged (wealthiest/tertiary education) – % coverage in most disadvantaged (least wealthy/primary or lower education).

Diff > 0 indicates most advantaged group reported higher coverage of RMNCH services.

Ratio = % coverage in most advantaged / % coverage in most disadvantaged. Ratio > 1 indicates most advantaged group were more likely to receive the RMNCH services.

Dark green indicates smaller inequality, red indicates higher inequality, grey indicates data not available/applicable.

***(Lower-middle-income countries, con’t)***

ANC — antenatal care, PNC — postnatal care, CI — confidence interval

Difference (Diff) = % coverage in most advantaged (wealthiest/tertiary education) – % coverage in most disadvantaged (least wealthy/primary or lower education).

Diff > 0 indicates most advantaged group reported higher coverage of RMNCH services.

Ratio = % coverage in most advantaged / % coverage in most disadvantaged. Ratio > 1 indicates most advantaged group were more likely to receive the RMNCH services.

Dark green indicates smaller inequality, red indicates higher inequality, grey indicates data not available/applicable.

**Appendix 10-3. Simple measures of socioeconomic inequality based on wealth index**

***(Low-income countries)***

ANC — antenatal care, PNC — postnatal care, CI — confidence interval

Difference (Diff) = % coverage in most advantaged (wealthiest/tertiary education) – % coverage in most disadvantaged (least wealthy/primary or lower education).

Diff > 0 indicates most advantaged group reported higher coverage of RMNCH services.

Ratio = % coverage in most advantaged / % coverage in most disadvantaged. Ratio > 1 indicates most advantaged group were more likely to receive the RMNCH services.

Dark green indicates smaller inequality, red indicates higher inequality, grey indicates data not available/applicable.

***(Low-income countries, con’t)***

ANC — antenatal care, PNC — postnatal care, CI — confidence interval

Difference (Diff) = % coverage in most advantaged (wealthiest/tertiary education) – % coverage in most disadvantaged (least wealthy/primary or lower education).

Diff > 0 indicates most advantaged group reported higher coverage of RMNCH services.

Ratio = % coverage in most advantaged / % coverage in most disadvantaged. Ratio > 1 indicates most advantaged group were more likely to receive the RMNCH services.

Dark green indicates smaller inequality, red indicates higher inequality, grey indicates data not available/applicable.

**Appendix 11-1. Simple measures of socioeconomic related inequality based on educational attainment, by countries**

***(Upper-middle-income countries)***

ANC — antenatal care, PNC — postnatal care, CI — confidence interval

Difference (Diff) = % coverage in most advantaged (wealthiest/tertiary education) – % coverage in most disadvantaged (least wealthy/primary or lower education).

Diff > 0 indicates most advantaged group reported higher coverage of RMNCH services.

Ratio = % coverage in most advantaged / % coverage in most disadvantaged. Ratio > 1 indicates most advantaged group were more likely to receive the RMNCH services.

Dark green indicates smaller inequality, red indicates higher inequality, grey indicates data not available/applicable.

**Appendix 11-2. Simple measures of socioeconomic related inequality based on educational attainment, by countries**

***(Lower-middle-income countries)***

ANC — antenatal care, PNC — postnatal care, CI — confidence interval

Difference (Diff) = % coverage in most advantaged (wealthiest/tertiary education) – % coverage in most disadvantaged (least wealthy/primary or lower education).

Diff > 0 indicates most advantaged group reported higher coverage of RMNCH services.

Ratio = % coverage in most advantaged / % coverage in most disadvantaged. Ratio > 1 indicates most advantaged group were more likely to receive the RMNCH services.

Dark green indicates smaller inequality, red indicates higher inequality, grey indicates data not available/applicable.

***(Lower-middle-income countries, con’t)***

ANC — antenatal care, PNC — postnatal care, CI — confidence interval

Difference (Diff) = % coverage in most advantaged (wealthiest/tertiary education) – % coverage in most disadvantaged (least wealthy/primary or lower education).

Diff > 0 indicates most advantaged group reported higher coverage of RMNCH services.

Ratio = % coverage in most advantaged / % coverage in most disadvantaged. Ratio > 1 indicates most advantaged group were more likely to receive the RMNCH services.

Dark green indicates smaller inequality, red indicates higher inequality, grey indicates data not available/applicable.

**Appendix 11-3. Simple measures of socioeconomic related inequality based on educational attainment, by countries**

***(Low-income countries)***

ANC — antenatal care, PNC — postnatal care, CI — confidence interval

Difference (Diff) = % coverage in most advantaged (wealthiest/tertiary education) – % coverage in most disadvantaged (least wealthy/primary or lower education).

Diff > 0 indicates most advantaged group reported higher coverage of RMNCH services.

Ratio = % coverage in most advantaged / % coverage in most disadvantaged. Ratio > 1 indicates most advantaged group were more likely to receive the RMNCH services.

Dark green indicates smaller inequality, red indicates higher inequality, grey indicates data not available/applicable.

***(Low-income countries, con’t)***

ANC — antenatal care, PNC — postnatal care, CI — confidence interval

Difference (Diff) = % coverage in most advantaged (wealthiest/tertiary education) – % coverage in most disadvantaged (least wealthy/primary or lower education).

Diff > 0 indicates most advantaged group reported higher coverage of RMNCH services.

Ratio = % coverage in most advantaged / % coverage in most disadvantaged. Ratio > 1 indicates most advantaged group were more likely to receive the RMNCH services.

Dark green indicates smaller inequality, red indicates higher inequality, grey indicates data not available/applicable.

**Appendix 12. Socioeconomic inequality of RMNCH services based on women’s educational attainment, by income group**

| **Outcomes** | **Overall** | | **Low-income**  **countries** | | **Lower-middle-income**  **countries** | | **Upper-middle-income**  **countries** | |
| --- | --- | --- | --- | --- | --- | --- | --- | --- |
|  | RII | (95% CI) | RII | (95% CI) | RII | (95% CI) | RII | (95% CI) |
| **Family Planning** |  |  |  |  |  |  |  |  |
| Contact | 1·26 | (1·24–1·29) | 1·19 | (1·16–1·23) | 1·33 | (1·29–1·37) | 1·14 | (1·08–1·21) |
| Crude | 1·41 | (1·36–1·47) | 1·41 | (1·33–1·49) | 1·56 | (1·48–1·64) | 1·02 | (0·92–1·14) |
| Quality | 1·77 | (1·66–1·89) | 1·5 | (1·36–1·66) | 2·19 | (1·98–2·42) | 1·25 | (1·04–1·51) |
| **ANC** |  |  |  |  |  |  |  |  |
| Contact | 1·40 | (1·38–1·41) | 1·2 | (1·18–1·23) | 1·51 | (1·49–1·53) | 1·07 | (1·04–1·10) |
| Crude | 1·91 | (1·87–1·95) | 1·58 | (1·49–1·67) | 2·27 | (2·21–2·33) | 1·18 | (1·13–1·22) |
| Quality | 2·44 | (2·37–2·52) | 1·98 | (1·84–2·14) | 3·11 | (3·01–3·22) | 1·27 | (1·20–1·34) |
| User-adherence | 3·01 | (2·88–3·15) | 2·02 | (1·84–2·23) | 3·92 | (3·73–4·13) | 1·16 | (1·02–1·33) |
| **Delivery** |  |  |  |  |  |  |  |  |
| Contact | 1·52 | (1·50–1·54) | 1·43 | (1·38–1·47) | 1·64 | (1·61–1·66) | 1·05 | (1·03–1·06) |
| Crude | 1·57 | (1·55–1·60) | 1·41 | (1·36–1·45) | 1·74 | (1·71–1·77) | 1·05 | (1·03–1·07) |
| **PNC** |  |  |  |  |  |  |  |  |
| Contact | 1·43 | (1·41–1·45) | 1·31 | (1·28–1·34) | 1·52 | (1·49–1·54) | 1·01 | (1·00–1·02) |
| Crude | 1·66 | (1·60–1·71) | 1·32 | (1·24–1·41) | 1·96 | (1·88–2·05) | 1·12 | (1·07–1·18) |
| Quality | 2·23 | (2·11–2·36) | 1·61 | (1·47–1·76) | 2·99 | (2·77–3·23) | 1·09 | (0·93–1·28) |
| **Immunisation** |  |  |  |  |  |  |  |  |
| Contact | 1·25 | (1·24–1·27) | 1·18 | (1·15–1·21) | 1·29 | (1·27–1·31) | 1·04 | (0·98–1·11) |
| Crude | 1·42 | (1·39–1·45) | 1·28 | (1·22–1·33) | 1·5 | (1·47–1·54) | 1·04 | (0·95–1·14) |
| Quality | 1·52 | (1·49–1·56) | 1·36 | (1·30–1·42) | 1·62 | (1·58–1·67) | 1·04 | (0·92–1·18) |
| User-adherence | 2·19 | (2·08–2·30) | 1·55 | (1·43–1·68) | 2·65 | (2·48–2·82) | 0·89 | (0·68–1·17) |
| **Diarrhoea treatment** |  |  |  |  |  |  |  |  |
| Contact | 1·14 | (1·10–1·17) | 1·27 | (1·20–1·35) | 1·09 | (1·06–1·13) | 0·97 | (0·75–1·25) |
| Crude | 1·31 | (1·25–1·37) | 1·3 | (1·20–1·41) | 1·33 | (1·26–1·39) | 1·18 | (0·85–1·64) |
| Quality | 1·31 | (1·24–1·37) | 1·28 | (1·16–1·40) | 1·33 | (1·26–1·41) | 1·15 | (0·78–1·69) |
| User-adherence | 1·35 | (1·23–1·48) | 1·35 | (1·15–1·58) | 1·38 | (1·23–1·55) | 0·86 | (0·42–1·77) |
| **Use of insecticide-treated nets^†^** |  |  |  |  |  |  |  |  |
| Contact | 1·13 | (1·11–1·16) | 1·21 | (1·18–1·24) | 1·05 | (1·01–1·09) |  |  |
| Crude | 1·14 | (1·10–1·18) | 1·28 | (1·24–1·33) | 1·00 | (0·95–1·05) |  |  |

Notes:

RII — relative index of inequality, ANC — Antenatal care, PNC — Postnatal care

RII was estimated based on educational attainment
*38 countries: all countries except Colombia
†14 countries: malaria endemic countries only (see Appendix 1)

**Appendix 13. Relative index of inequality of RMNCH services, by educational attainment**

| **Income group** | **Countries** | **Family Planning** | |  | **Antenatal care** | |  | **Delivery care** | |  | **Postnatal care** | |  | **Immunisation** | |  | **Diarrhoea treatment** | |  | **Use of insecticide-treated nets** | |
| --- | --- | --- | --- | --- | --- | --- | --- | --- | --- | --- | --- | --- | --- | --- | --- | --- | --- | --- | --- | --- | --- |
|  |  | **Contact** | **Quality** |  | **Contact** | **Quality** |  | **Contact** | **Crude** |  | **Contact** | **Quality** |  | **Contact** | **Quality** |  | **Contact** | **Quality** |  | **Contact** | **Crude** |
|  |  | **RII (95% CI)** | |  | **RII (95% CI)** | |  | **RII (95% CI)** | |  | **RII (95% CI)** | |  | **RII (95% CI)** | |  | **RII (95% CI)** | |  | **RII (95% CI)** | |
| **Upper-middle-income** | Albania | 1·55 | 1·59 |  | 1·15 | 2·2 |  | 1·01 | 0·98 |  | 1 | 1·02 |  | 0·92 | 0·89 |  | 1·2 | 1·7 |  |  |  |
|  |  | (1·30–1·85) | (0·11–23·13) |  | (1·04–1·27) | (1·70–2·85) |  | (0·99–1·04) | (0·93–1·02) |  | (1·00–1·01) | (0·80–1·32) |  | (0·78–1·09) | (0·59–1·34) |  | (0·67–2·14) | (0·69–4·19) |  |  |  |
|  | Colombia | 1·28 | 1·31 |  | 1·05 | 1·03 |  | 1·03 | 1·04 |  |  |  |  |  |  |  |  |  |  |  |  |
|  |  | (1·17–1·39) | (0·98–1·77) |  | (1·01–1·08) | (1·00–1·05) |  | (1·01–1·06) | (1·01–1·07) |  |  |  |  |  |  |  |  |  |  |  |  |
|  | Jordan | 0·97 | 0·73 |  | 1·03 | 1·18 |  | 1·01 | 1·02 |  | 1 | 0·93 |  | 1·06 | 0·98 |  | 1·19 | 1·67 |  |  |  |
|  |  | (0·84–1·12) | (0·52–1·04) |  | (1·00–1·05) | (1·06–1·31) |  | (1·00–1·01) | (1·00–1·04) |  | (1·00–1·01) | (0·66–1·33) |  | (0·97–1·15) | (0·85–1·13) |  | (0·84–1·67) | (0·88–3·19) |  |  |  |
|  | Maldives | 0·98 | 0·75 |  | 0·92 | 0·87 |  | 0·99 | 0·95 |  | 1 | 1·01 |  | 0·95 | 0·88 |  | 0·93 | 1·26 |  |  |  |
|  |  | (0·93–1·03) | (0·18–3·19) |  | (0·81–1·05) | (0·72–1·04) |  | (0·98–1·00) | (0·91–1·00) |  | (0·99–1·00) | (0·81–1·26) |  | (0·84–1·08) | (0·73–1·07) |  | (0·72–1·20) | (0·45–3·52) |  |  |  |
|  | South Africa | 1·04 | 0·85 |  | 1·07 | 1·23 |  | 1·06 | 1·08 |  | 1·01 | 1·27 |  | 1·09 | 1·09 |  | 1·59 | 2·02 |  |  |  |
|  |  | (0·91–1·18) | (0·52–1·39) |  | (0·97–1·17) | (1·06–1·43) |  | (1·01–1·11) | (1·02–1·14) |  | (0·99–1·03) | (0·91–1·76) |  | (0·93–1·27) | (0·78–1·52) |  | (1·05–2·40) | (1·08–3·77) |  |  |  |
|  |  |  |  |  |  |  |  |  |  |  |  |  |  |  |  |  |  |  |  |  |  |
|  | Angola | 2·82 | 170·6 |  | 1·63 | 5·53 |  | 3·83 | 4·95 |  | 2·63 | 14·57 |  | 2·06 | 4·77 |  | 1·47 | 1·6 |  | 1·15 | 1·2 |
|  |  | (2·24–3·54) | (37·75–770·87) |  | (1·48–1·79) | (4·48–6·83) |  | (3·20–4·58) | (4·01–6·12) |  | (2·26–3·05) | (7·04–30·2) |  | (1·71–2·47) | (3·28–6·93) |  | (1·01–2·13) | (1·01–2·53) |  | (0·91–1·45) | (0·91–1·59) |
| **Lower-middle-income** | Armenia | 1·62 | 3·34 |  | 1·04 | 1·27 |  | 1·00 | 1·01 |  | 1·00 | 1·02 |  | 1·03 | 0·76 |  | 1·11 | 1·07 |  |  |  |
|  |  | (1·37–1·92) | (1·32–8·41) |  | (0·99–1·09) | (0·83–1·96) |  | (0.99–1·01) | (1·00–1·02) |  | (0·87–1·17) | (0·87–1·21) |  | (0·97–1·10) | (0·59–0·98) |  | (0·22–5·67) | (0·14–8·32) |  |  |  |
|  | Bangladesh | 0·77 |  |  | 2·68 | 19·21 |  | 4·34 | 5·15 |  | 3·75 | 7·93 |  | 1·06 | 1·42 |  | 1·17 | 0·96 |  |  |  |
|  |  | (0·65–0·90) |  |  | (2·34–3·06) | (12·89–28·63) |  | (3·57–5·28) | (4·07–6·53) |  | (3·14–4·48) | (3·00–21·0) |  | (1·02–1·10) | (1·26–1·61) |  | (0·64–2·15) | (0·47–1·96) |  |  |  |
|  | Cameroon | 1·21 | 2·24 |  | 1·46 | 3·24 |  | 2·86 | 2·91 |  | 2·59 | 4·95 |  | 1·32 | 1·82 |  | 0·92 | 1·8 |  | 0·99 | 0·84 |
|  |  | (1·05–1·39) | (0·98–5·13) |  | (1·35–1·59) | (2·78–3·77) |  | (2·46–3·33) | (2·47–3·43) |  | (2·26–2·98) | (3·46–7·08) |  | (1·17–1·49) | (1·43–2·31) |  | (0·67–1·27) | (0·76–4·25) |  | (0·91–1·08) | (0·75–0·94) |
|  | Ghana | 1·12 | 0·87 |  | 1·1 | 1·61 |  | 2·1 | 2·1 |  | 1·24 | 3·24 |  | 1·07 | 1·17 |  | 0·97 | 1·33 |  | 1·02 | 0·68 |
|  |  | (0·90–1·40) | (0·47–1·58) |  | (1·04–1·16) | (1·39–1·86) |  | (1·76–2·50) | (1·76–2·52) |  | (1·09–1·40) | (1·24–8·47) |  | (1·01–1·14) | (0·99–1·39) |  | (0·72–1·31) | (0·76–2·32) |  | (0·92–1·14) | (0·55–0·85) |
|  | Guatemala | 0·98 | 2·24 |  | 1·17 | 2·47 |  | 2·62 | 2·62 |  | 1·1 | 0·45 |  | 1·01 | 1·36 |  | 1·01 | 1·35 |  |  |  |
|  |  | (0·92–1·05) | (1·64–3·06) |  | (1·11–1·22) | (2·16–2·84) |  | (2·34–2·94) | (2·33–2·94) |  | (1·07–1·14) | (0·19–1·05) |  | (0·97–1·04) | (1·15–1·60) |  | (0·87–1·17) | (1·07–1·70) |  |  |  |
|  | India | 1·27 | 1·18 |  | 1·68 | 3·31 |  | 1·49 | 1·61 |  | 1·43 | 3·06 |  | 1·17 | 1·42 |  | 1·19 | 1·5 |  |  |  |
|  |  | (1·16–1·38) | (0·73–1·91) |  | (1·65–1·71) | (3·16–3·45) |  | (1·47–1·52) | (1·58–1·64) |  | (1·40–1·45) | (2·75–3·42) |  | (1·14–1·19) | (1·37–1·46) |  | (1·14–1·23) | (1·38–1·63) |  |  |  |
|  | Indonesia | 1·29 | 1·78 |  | 1·1 | 2·42 |  | 1·32 | 1·63 |  | 1·32 | 2·44 |  | 1·12 | 1·34 |  | 0·99 | 1·01 |  |  |  |
|  |  | (1·21–1·37) | (1·43–2·21) |  | (1·07–1·12) | (2·03–2·89) |  | (1·26–1·38) | (1·53–1·74) |  | (1·26–1·37) | (1·49–4·02) |  | (1·05–1·19) | (1·19–1·50) |  | (0·88–1·10) | (0·77–1·32) |  |  |  |
|  | Kenya | 1·13 | 2·26 |  | 1·13 | 1·87 |  | 3·05 | 3·19 |  | 2·19 | 3·77 |  | 1·07 | 1·43 |  | 0·88 | 0·85 |  | 1·46 | 1·7 |
|  |  | (1·04–1·23) | (1·64–3·12) |  | (1·10–1·16) | (1·65–2·12) |  | (2·77–3·36) | (2·88–3·53) |  | (2·03–2·35) | (2·46–5·77) |  | (1·03–1·10) | (1·32–1·55) |  | (0·75–1·03) | (0·70–1·04) |  | (1·36–1·57) | (1·54–1·87) |
|  | Lesotho | 1 | 1·6 |  | 1·09 | 1·79 |  | 1·63 | 1·72 |  | 1·17 | 2·33 |  | 1·05 | 1·11 |  | 0·92 | 0·47 |  |  |  |
|  |  | (0·86–1·15) | (0·82–3·13) |  | (1·03–1·15) | (1·39–2·30) |  | (1·47–1·80) | (1·54–1·91) |  | (1·10–1·23) | (1·30–4·18) |  | (1·01–1·10) | (0·90–1·37) |  | (0·56–1·52) | (0·25–0·88) |  |  |  |
|  | Myanmar | 1·64 | 1·65 |  | 1·44 | 1·9 |  | 2·44 | 3·28 |  | 2·09 | 4·31 |  | 1·29 | 2·11 |  | 1·29 | 0·94 |  |  |  |
|  |  | (1·42–1·89) | (1·00–2·74) |  | (1·27–1·62) | (1·90–1·90) |  | (2·02–2·96) | (2·46–4·37) |  | (1·77–2·47) | (0·05–339·50) |  | (1·08–1·54) | (1·55–2·88) |  | (0·95–1·75) | (0·70–1·26) |  |  |  |
|  | Nigeria | 1·16 | 4 |  | 2·36 | 3·74 |  | 7·3 | 8·18 |  | 5·69 | 70·79 |  | 2·65 | 4·42 |  | 1·23 | 1·74 |  | 0·8 | 0·65 |
|  |  | (1·03–1·30) | (2·68–5·96) |  | (2·13–2·60) | (3·21–4·35) |  | (6·21–8·58) | (6·97–9·61) |  | (4·92–6·57) | (47·17–106·23) |  | (2·33–3·00) | (3·64–5·37) |  | (1·06–1·42) | (1·33–2·27) |  | (0·75–0·87) | (0·59–0·72) |
|  | Pakistan | 1·13 | 1·16 |  | 1·52 | 9·85 |  | 2·03 | 2·2 |  | 1·87 | 30·54 |  | 1·49 | 2·43 |  | 1·19 | 0·96 |  |  |  |
|  |  | (1·05–1·23) | (0·37–3·59) |  | (1·41–1·64) | (7·23–13·42) |  | (1·77–2·34) | (1·90–2·53) |  | (1·65–2·12) | (14·34–65·05) |  | (1·32–1·68) | (2·00–2·95) |  | (1·00–1·41) | (0·65–1·41) |  |  |  |
|  | Papua New Guinea | 1·59 | 3·02 |  | 1·8 | 3·7 |  | 3·16 | 3·47 |  | 2·53 | 9·99 |  | 2·44 | 3·37 |  | 1·37 | 1·87 |  |  |  |
|  |  | (1·34–1·89) | (1·73–5·29) |  | (1·58–2·04) | (2·64–5·20) |  | (2·65–3·77) | (2·87–4·19) |  | (2·19–2·92) | (6·18–16·13) |  | (1·97–3·03) | (2·39–4·75) |  | (0·82–2·28) | (0·99–3·54) |  |  |  |
|  | Philippines | 0·73 | 0·52 |  | 1·21 | 2·73 |  | 1·51 | 1·68 |  | 1·3 | 1·77 |  | 1·27 | 1·47 |  | 1·14 | 1·17 |  |  |  |
|  |  | (0·62–0·87) | (0·40–0·68) |  | (1·12–1·30) | (2·31–3·24) |  | (1·36–1·66) | (1·51–1·87) |  | (1·19–1·42) | (1·40–2·25) |  | (1·16–1·39) | (1·26–1·72) |  | (0·72–1·81) | (0·68–2·00) |  |  |  |
|  | Timor-Leste | 1·26 | 1·17 |  | 1·35 | 2·55 |  | 3·06 | 4·21 |  | 2·28 | 10·87 |  | 1·51 | 1·93 |  | 1·31 | 1·3 |  |  |  |
|  |  | (1·14–1·40) | (0·74–1·86) |  | (1·25–1·45) | (1·90–3·44) |  | (2·60–3·60) | (3·45–5·14) |  | (2·00–2·59) | (6·24–18·94) |  | (1·31–1·73) | (1·50–2·50) |  | (0·94–1·83) | (0·90–1·88) |  |  |  |
|  | Zambia | 1·04 | 1·15 |  | 1·08 | 1·84 |  | 1·36 | 1·35 |  | 1·2 | 2·08 |  | 1·03 | 1·18 |  | 0·95 | 1·06 |  |  |  |
|  |  | (0·92–1·18) | (0·87–1·51) |  | (1·04–1·12) | (1·46–2·32) |  | (1·23–1·49) | (1·23–1·49) |  | (1·13–1·29) | (1·56–2·77) |  | (1·00–1·07) | (1·08–1·30) |  | (0·78–1·17) | (0·85–1·32) |  |  |  |
|  |  |  |  |  |  |  |  |  |  |  |  |  |  |  |  |  |  |  |  |  |  |
| **Low-income** | Afghanistan | 1·27 | 2·30 |  | 1·48 | 6·57 |  | 3·51 | 3·86 |  | 2·67 | 2·2 |  | 1·3 | 1·51 |  | 1·07 | 1·05 |  |  |  |
|  |  | (1·09–1·48) | (1·27–4·14) |  | (1·25–1·76) | (3·30–13·11) |  | (2·98–4·13) | (3·26–4·58) |  | (2·32–3·06) | (0·85–5·68) |  | (1·10–1·53) | (1·17–1·95) |  | (0·89–1·28) | (0·82–1·36) |  |  |  |
|  | Benin | 1·23 | 2·22 |  | 1·51 | 3·56 |  | 1·76 | 1·79 |  | 1·61 | 2·82 |  | 1·46 | 1·74 |  | 1·24 | 1·6 |  | 1·1 | 1·14 |
|  |  | (1·06–1·42) | (1·18–4·21) |  | (1·39–1·65) | (3·11–4·08) |  | (1·61–1·93) | (1·62–1·96) |  | (1·49–1·75) | (2·31–3·46) |  | (1·31–1·62) | (1·49–2·04) |  | (0·91–1·68) | (0·99–2·57) |  | (1·05–1·15) | (1·07–1·21) |
|  | Burundi | 1·04 | 0·85 |  | 1·02 | 3·5 |  | 1·21 | 1·22 |  | 1·18 | 2·05 |  | 1·01 | 1·01 |  | 1·13 | 1·24 |  | 1·97 | 2·07 |
|  |  | (1·00–1·08) | (0·53–1·35) |  | (1·00–1·03) | (2·27–5·41) |  | (1·15–1·27) | (1·16–1·29) |  | (1·13–1·23) | (1·66–2·53) |  | (0·99–1·03) | (0·96–1·07) |  | (0·98–1·30) | (0·96–1·60) |  | (1·78–2·18) | (1·85–2·33) |
|  | Cambodia | 1·00 | 0·69 |  | 1·12 | 1·61 |  | 1·31 | 1·41 |  | 1·3 | 1·5 |  | 1·18 | 1·82 |  | 0·87 | 0·58 |  |  |  |
|  |  | (0·87–1·15) | (0·53–0·89) |  | (1·07–1·16) | (1·20–2·16) |  | (1·21–1·42) | (1·28–1·55) |  | (1·20–1·40) | (1·20–1·87) |  | (1·11–1·26) | (1·55–2·15) |  | (0·67–1·11) | (0·32–1·04) |  |  |  |
|  | Chad | 1·12 | 2·22 |  | 1·09 | 2·62 |  | 1·51 | 1·9 |  | 1·35 | 0·65 |  | 1·25 | 1·59 |  | 1·39 | 2·13 |  | 1·07 | 0·99 |
|  |  | (0·92–1·37) | (0·77–6·38) |  | (0·94–1·26) | (1·80–3·82) |  | (1·18–1·92) | (1·41–2·56) |  | (1·08–1·70) | (0·07–5·68) |  | (1·01–1·55) | (1·13–2·23) |  | (1·04–1·86) | (1·26–3·61) |  | (1·03–1·11) | (0·89–1·10) |
|  | Ethiopia | 1·45 | 3·90 |  | 1·86 | 5·14 |  | 4·66 | 4·85 |  | 4·66 | 487·5 |  | 1·46 | 2·62 |  | 1·46 | 1·59 |  |  |  |
|  |  | (1·22–1·72) | (2·09–7·30) |  | (1·53–2·28) | (3·04–8·68) |  | (3·12–6·97) | (3·20–7·35) |  | (3·22–6·75) | (28·4–8,367) |  | (1·14–1·86) | (1·71–3·99) |  | (0·96–2·23) | (0·75–3·34) |  |  |  |
|  | Guinea | 2·18 | 6·09 |  | 1·32 | 7·01 |  | 2·98 | 3·29 |  | 2·24 | 4·03 |  | 1·8 | 3·08 |  | 1·87 | 2·43 |  | 0·91 | 1·07 |
|  |  | (1·54–3·09) | (1·34–27·71) |  | (1·16–1·52) | (5·01–9·81) |  | (2·38–3·72) | (2·54–4·27) |  | (1·87–2·69) | (2·57–6·30) |  | (1·38–2·36) | (1·61–5·88) |  | (1·42–2·45) | (1·79–3·31) |  | (0·77–1·08) | (0·82–1·40) |
|  | Haiti | 1·35 | 0·70 |  | 1·31 | 3·11 |  | 6·8 | 6·93 |  | 2·35 | 16·77 |  | 1·37 | 2·59 |  | 1·21 | 1·25 |  |  |  |
|  |  | (1·19–1·54) | (0·41–1·20) |  | (1·22–1·40) | (2·58–3·75) |  | (5·37–8·60) | (5·45–8·82) |  | (2·02–2·72) | (8·27–34·01) |  | (1·19–1·59) | (1·85–3·63) |  | (0·83–1·77) | (0·75–2·09) |  |  |  |
|  | Malawi | 1·01 | 1·08 |  | 1·03 | 1·6 |  | 1·1 | 1·1 |  | 1·06 | 1·36 |  | 1·02 | 1·11 |  | 1·07 | 1·11 |  | 1·48 | 1·59 |
|  |  | (0·94–1·08) | (0·94–1·23) |  | (1·01–1·06) | (1·24–2·05) |  | (1·06–1·14) | (1·06–1·14) |  | (1·04–1·09) | (1·15–1·61) |  | (0·99–1·05) | (1·03–1·18) |  | (0·95–1·21) | (0·95–1·30) |  | (1·38–1·59) | (1·44–1·77) |
|  | Mali | 1·45 | 5·26 |  | 1·51 | 6·31 |  | 2·37 | 2·41 |  | 2·08 | 22·38 |  | 1·28 | 1·31 |  | 1·22 | 1·43 |  | 0·99 | 0·96 |
|  |  | (1·17–1·80) | (2·91–9·50) |  | (1·33–1·71) | (4·54–8·78) |  | (1·93–2·90) | (1·95–2·97) |  | (1·73–2·50) | (8·48–59·06) |  | (1·09–1·49) | (1·00–1·70) |  | (0·90–1·67) | (0·79–2·60) |  | (0·96–1·03) | (0·90–1·01) |
|  | Nepal | 0·92 | 0·41 |  | 1·22 | 2·83 |  | 2·09 | 2·14 |  | 1·72 | 4·53 |  | 1·04 | 1·07 |  | 1·26 | 0·94 |  |  |  |
|  |  | (0·85–1·00) | (0·29–0·58) |  | (1·11–1·34) | (2·34–3·43) |  | (1·76–2·48) | (1·79–2·55) |  | (1·52–1·96) | (2·46–8·35) |  | (0·98–1·10) | (0·95–1·22) |  | (0·86–1·86) | (0·47–1·88) |  |  |  |
|  | Rwanda | 1·26 | 1·20 |  | 1·01 | 1·29 |  | 1·15 | 1·16 |  | 1·11 | 1·49 |  | 1·03 | 1·35 |  | 1·52 | 1·79 |  |  |  |
|  |  | (1·17–1·34) | (0·99–1·46) |  | (1·00–1·02) | (1·00–1·66) |  | (1·10–1·20) | (1·11–1·21) |  | (1·07–1·15) | (1·03–2·16) |  | (1·00–1·05) | (1·05–1·75) |  | (1·15–2·01) | (1·07–3·01) |  |  |  |
|  | Senegal | 1·18 | 1·96 |  | 1·08 | 2·57 |  | 2·56 | 2·56 |  | 1·48 | 2·95 |  | 1·11 | 1·34 |  | 1·39 | 1·19 |  |  |  |
|  |  | (1·10–1·27) | (1·31–2·92) |  | (1·04–1·11) | (2·25–2·95) |  | (2·24–2·93) | (2·23–2·93) |  | (1·38–1·58) | (2·23–3·90) |  | (1·05–1·17) | (1·19–1·50) |  | (1·09–1·77) | (0·82–1·74) |  |  |  |
|  | Tajikistan | 1·21 | 1·77 |  | 1·12 | 3·54 |  | 1·09 | 1·2 |  | 1·05 | 1·56 |  | 1·03 | 0·92 |  | 1·08 | 1 |  |  |  |
|  |  | (1·05–1·40) | (1·14–2·73) |  | (1·05–1·20) | (2·55–4·91) |  | (1·03–1·15) | (1·10–1·31) |  | (1·01–1·09) | (1·25–1·94) |  | (0·95–1·11) | (0·79–1·08) |  | (0·73–1·59) | (0·57–1·72) |  |  |  |
|  | Tanzania | 1·08 | 1·49 |  | 1·02 | 4·87 |  | 2·2 | 2·25 |  | 1·83 | 3·97 |  | 1·09 | 1·36 |  | 1·08 | 1·29 |  | 1·31 | 1·35 |
|  |  | (0·97–1·20) | (1·03–2·17) |  | (0·99–1·04) | (3·63–6·55) |  | (1·88–2·56) | (1·89–2·67) |  | (1·61–2·09) | (2·71–5·82) |  | (1·04–1·13) | (1·18–1·57) |  | (0·87–1·34) | (0·86–1·94) |  | (1·16–1·48) | (1·16–1·56) |
|  | Uganda | 0·97 | 1·17 |  | 1·01 | 3·27 |  | 1·45 | 1·47 |  | 1·31 | 1 |  | 1 | 1·11 |  | 0·91 | 0·97 |  | 1·25 | 1·28 |
|  |  | (0·91–1·04) | (0·91–1·50) |  | (0·99–1·04) | (2·56–4·16) |  | (1·35–1·57) | (1·36–1·60) |  | (1·22–1·40) | (0·78–1·27) |  | (0·96–1·04) | (0·99–1·25) |  | (0·81–1·03) | (0·77–1·22) |  | (1·18–1·32) | (1·18–1·39) |
|  | Zimbabwe | 1·27 | 2·23 |  | 1·14 | 2·33 |  | 1·51 | 1·53 |  | 1·24 | 2·08 |  | 1·08 | 1·24 |  | 1·55 | 2·04 |  | 1·09 | 0·88 |
|  |  | (1·08–1·48) | (1·56–3·18) |  | (1·05–1·25) | (1·78–3·04) |  | (1·32–1·72) | (1·34–1·76) |  | (1·13–1·37) | (1·61–2·67) |  | (0·93–1·26) | (0·98–1·56) |  | (0·95–2·54) | (1·17–3·55) |  | (1·01–1·18) | (0·77–1·01) |

**Notes:**

RII — relative index of inequality

RII > 1 indicates higher inequality (more educated groups are more likely to receive services compared to less educated groups).

Dark green indicates smaller inequality, red indicates higher inequality, grey indicates data not available.

**Appendix 14. Slope index of inequality of RMNCH services, by socioeconomic status**

| **Income group** | **Countries** | **Family Planning** | |  | **Antenatal care** | |  | **Delivery care** | |  | **Postnatal care** | |  | **Immunisation** | |  | **Diarrhoea treatment** | |  | **Use of insecticide-treated nets** | |
| --- | --- | --- | --- | --- | --- | --- | --- | --- | --- | --- | --- | --- | --- | --- | --- | --- | --- | --- | --- | --- | --- |
|  |  | **Contact** | **Quality** |  | **Contact** | **Quality** |  | **Contact** | **Crude** |  | **Contact** | **Quality** |  | **Contact** | **Quality** |  | **Contact** | **Quality** |  | **Contact** | **Crude** |
|  |  | **SII (95% CI)** | |  | **SII (95% CI)** | |  | **SII (95% CI)** | |  | **SII (95% CI)** | |  | **SII (95% CI)** | |  | **SII (95% CI)** | |  | **SII (95% CI)** | |
| **Upper-middle-income** | Albania | 0·22 | 0 |  | 0·12 | 0·44 |  | 0·01 | -0·02 |  | 0 | 0·01 |  | -0·08 | -0·08 |  | 0·09 | 0·04 |  |  |  |
|  |  | (0·14–0·31) | (-0·01–0·01) |  | (0·03–0·21) | (0·28–0·59) |  | (-0·01–0·04) | (-0·07–0·02) |  | (-0·00–0·01) | (-0·17–0·19) |  | (-0·23–0·08) | (-0·44–0·29) |  | (-0·26–0·44) | (-0·39–0·47) |  |  |  |
|  | Colombia | 0·17 | 0·07 |  | 0·05 | 0·09 |  | 0·04 | 0·04 |  |  |  |  |  |  |  |  |  |  |  |  |
|  |  | (0·11–0·23) | (0·00–0·14) |  | (0·01–0·08) | (0·02–0·16) |  | (0·01–0·06) | (0·01–0·07) |  |  |  |  |  |  |  |  |  |  |  |  |
|  | Jordan | -0·02 | -0·03 |  | 0·02 | 0·12 |  | 0·01 | 0·02 |  | 0 | -0·02 |  | 0·05 | -0·02 |  | 0·09 | 0·11 |  |  |  |
|  |  | (-0·09–0·06) | (-0·07–0·01) |  | (0·00–0·05) | (0·05–0·19) |  | (-0·00–0·01) | (-0·00–0·04) |  | (-0·00–0·01) | (-0·08–0·05) |  | (-0·03–0·13) | (-0·13–0·10) |  | (-0·09–0·26) | (-0·04–0·26) |  |  |  |
|  | Maldives | -0·02 | 0 |  | -0·07 | -0·09 |  | -0·01 | -0·05 |  | 0 | 0·01 |  | -0·04 | -0·1 |  | -0·06 | -0·02 |  |  |  |
|  |  | (-0·07–0·03) | (-0·00–-0·00) |  | (-0·18–0·05) | (-0·22–0·03) |  | (-0·02–0·00) | (-0·09–-0·00) |  | (-0·01–0·00) | (-0·12–0·13) |  | (-0·15–0·07) | (-0·25–0·05) |  | (-0·30–0·18) | (-0·49–0·44) |  |  |  |
|  | South Africa | 0·02 | -0·05 |  | 0·06 | 0·16 |  | 0·06 | 0·08 |  | 0·01 | 0·1 |  | 0·08 | 0·06 |  | 0·34 | 0·36 |  |  |  |
|  |  | (-0·07–0·12) | (-0·12–0·02) |  | (-0·03–0·15) | (0·05–0·27) |  | (0·01–0·11) | (0·02–0·13) |  | (-0·01–0·03) | (-0·04–0·23) |  | (-0·06–0·22) | (-0·17–0·28) |  | (0·05–0·62) | (0·05–0·66) |  |  |  |
|  |  |  |  |  |  |  |  |  |  |  |  |  |  |  |  |  |  |  |  |  |  |
|  | Angola | 0·46 | 0·18 |  | 0·4 | 0·68 |  | 0·58 | 0·6 |  | 0·54 | 0·15 |  | 0·49 | 0·26 |  | 0·19 | 0·21 |  | 0·07 | 0·07 |
|  |  | (0·36–0·56) | (0·18–0·18) |  | (0·32–0·48) | (0·59–0·77) |  | (0·49–0·67) | (0·51–0·70) |  | (0·46–0·63) | (0·15–0·15) |  | (0·37–0·60) | (0·26–0·26) |  | (0·02–0·36) | (0·04–0·37) |  | (-0·03–0·17) | (-0·02–0·16) |
| **Lower-middle-income** | Armenia | 0·28 | 0·04 |  | 0·04 | 0·09 |  | 0·00 | 0·01 |  | 0 | 0·02 |  | 0·03 | -0·26 |  | 0·03 | -0·05 |  |  |  |
|  |  | (0·19–0·37) | (0·04–0·04) |  | (-0·01–0·08) | (-0·07–0·26) |  | (-0·00–0·00) | (-0·00–0·02) |  | (0·00–0·00) | (-0·12–0·17) |  | (-0·03–0·10) | (-0·50–-0·01) |  | (-0·54–0·60) | (-0·66–0·56) |  |  |  |
|  | Bangladesh | -0·11 |  |  | 0·62 | 0·43 |  | 0·38 | 0·32 |  | 0·55 | 0·03 |  | 0·06 | 0·3 |  | 0·1 | -0·02 |  |  |  |
|  |  | (-0·17–-0·06) |  |  | (0·55–0·69) | (0·43–0·43) |  | (0·38–0·38) | (0·32–0·32) |  | (0·47–0·64) | (0·03–0·03) |  | (0·02–0·10) | (0·20–0·40) |  | (-0·27–0·47) | (-0·35–0·31) |  |  |  |
|  | Cameroon | 0·11 | 0·00 |  | 0·34 | 0·68 |  | 0·8 | 0·77 |  | 0·75 | 0·4 |  | 0·23 | 0·36 |  | -0·03 | 0·07 |  | -0·01 | -0·12 |
|  |  | (0·03–0·20) | (0·00–0·01) |  | (0·27–0·41) | (0·61–0·76) |  | (0·69–0·90) | (0·66–0·87) |  | (0·65–0·85) | (0·32–0·47) |  | (0·13–0·33) | (0·22–0·51) |  | (-0·20–0·14) | (-0·07–0·21) |  | (-0·08–0·06) | (-0·20–-0·05) |
|  | Ghana | 0·06 | -0·01 |  | 0·09 | 0·37 |  | 0·52 | 0·52 |  | 0·18 | 0·09 |  | 0·07 | 0·13 |  | -0·02 | 0·12 |  | 0·02 | -0·23 |
|  |  | (-0·06–0·18) | (-0·08–0·05) |  | (0·04–0·14) | (0·26–0·47) |  | (0·41–0·63) | (0·40–0·63) |  | (0·07–0·29) | (0·01–0·16) |  | (0·01–0·13) | (-0·01–0·27) |  | (-0·22–0·18) | (-0·08–0·31) |  | (-0·07–0·11) | (-0·35–-0·11) |
|  | Guatemala | -0·01 | 0·11 |  | 0·14 | 0·48 |  | 0·66 | 0·65 |  | 0·09 | -0·02 |  | 0·01 | 0·18 |  | 0·01 | 0·12 |  |  |  |
|  |  | (-0·07–0·04) | (0·07–0·14) |  | (0·09–0·18) | (0·41–0·54) |  | (0·59–0·72) | (0·58–0·72) |  | (0·06–0·12) | (-0·03–-0·00) |  | (-0·03–0·04) | (0·08–0·28) |  | (-0·09–0·10) | (0·03–0·21) |  |  |  |
|  | India | 0·11 | 0·01 |  | 0·42 | 0·5 |  | 0·34 | 0·39 |  | 0·31 | 0·11 |  | 0·14 | 0·25 |  | 0·13 | 0·17 |  |  |  |
|  |  | (0·07–0·14) | (-0·00–0·02) |  | (0·41–0·44) | (0·49–0·52) |  | (0·33–0·35) | (0·37–0·40) |  | (0·30–0·32) | (0·10–0·12) |  | (0·12–0·16) | (0·23–0·28) |  | (0·10–0·16) | (0·14–0·21) |  |  |  |
|  | Indonesia | 0·14 | 0·04 |  | 0·09 | 0·27 |  | 0·26 | 0·42 |  | 0·26 | 0·04 |  | 0·1 | 0·21 |  | -0·01 | 0·01 |  |  |  |
|  |  | (0·10–0·18) | (0·03–0·06) |  | (0·07–0·11) | (0·22–0·33) |  | (0·22–0·30) | (0·36–0·47) |  | (0·22–0·30) | (0·02–0·06) |  | (0·05–0·16) | (0·13–0·29) |  | (-0·10–0·08) | (-0·11–0·12) |  |  |  |
|  | Kenya | 0·09 | 0·14 |  | 0·11 | 0·27 |  | 0·67 | 0·68 |  | 0·55 | 0·1 |  | 0·06 | 0·29 |  | -0·08 | -0·08 |  | 0·28 | 0·33 |
|  |  | (0·02–0·15) | (0·08–0·20) |  | (0·09–0·14) | (0·21–0·32) |  | (0·62–0·72) | (0·63–0·73) |  | (0·50–0·60) | (0·07–0·13) |  | (0·03–0·10) | (0·22–0·35) |  | (-0·19–0·02) | (-0·18–0·02) |  | (0·23–0·33) | (0·28–0·39) |
|  | Lesotho | 0 | 0·07 |  | 0·08 | 0·28 |  | 0·4 | 0·43 |  | 0·15 | 0·1 |  | 0·05 | 0·08 |  | -0·07 | -0·35 |  |  |  |
|  |  | (-0·11–0·11) | (-0·01–0·15) |  | (0·02–0·13) | (0·15–0·40) |  | (0·31–0·48) | (0·35–0·51) |  | (0·09–0·20) | (0·03–0·17) |  | (0·01–0·09) | (-0·09–0·26) |  | (-0·34–0·21) | (-0·61–-0·09) |  |  |  |
|  | Myanmar | 0·27 | 0·04 |  | 0·29 | 0·34 |  | 0·57 | 0·32 |  | 0·49 | 0·01 |  | 0·22 | 0·42 |  | 0·16 | -0·02 |  |  |  |
|  |  | (0·19–0·34) | (-0·01–0·08) |  | (0·20–0·39) | (0·24–0·44) |  | (0·46–0·68) | (0·32–0·32) |  | (0·39–0·60) | (-0·01–0·02) |  | (0·05–0·39) | (0·23–0·60) |  | (-0·04–0·37) | (-0·22–0·17) |  |  |  |
|  | Nigeria | 0·08 | 0 |  | 0·54 | 0·48 |  | 0·72 | 0·62 |  | 0·57 | 0·12 |  | 0·6 | 0·37 |  | 0·13 | 0·16 |  | -0·16 | -0·27 |
|  |  | (0·02–0·13) | (0·00–0·00) |  | (0·48–0·60) | (0·44–0·53) |  | (0·72–0·72) | (0·62–0·62) |  | (0·57–0·57) | (0·10–0·14) |  | (0·52–0·67) | (0·37–0·37) |  | (0·03–0·22) | (0·08–0·25) |  | (-0·21–-0·11) | (-0·33–-0·21) |
|  | Pakistan | 0·11 | 0 |  | 0·37 | 0·59 |  | 0·5 | 0·53 |  | 0·47 | 0·01 |  | 0·35 | 0·62 |  | 0·12 | -0·02 |  |  |  |
|  |  | (0·04–0·18) | (-0·02–0·03) |  | (0·31–0·43) | (0·49–0·68) |  | (0·41–0·60) | (0·44–0·62) |  | (0·38–0·55) | (0·01–0·02) |  | (0·24–0·45) | (0·49–0·75) |  | (-0·00–0·25) | (-0·14–0·10) |  |  |  |
|  | Papua New Guinea | 0·27 | 0·08 |  | 0·44 | 0·3 |  | 0·65 | 0·66 |  | 0·63 | 0·37 |  | 0·58 | 0·48 |  | 0·11 | 0·16 |  |  |  |
|  |  | (0·17–0·38) | (0·04–0·13) |  | (0·35–0·53) | (0·22–0·38) |  | (0·56–0·74) | (0·58–0·75) |  | (0·54–0·72) | (0·30–0·43) |  | (0·45–0·72) | (0·36–0·59) |  | (-0·08–0·30) | (-0·00–0·31) |  |  |  |
|  | Philippines | -0·2 | -0·15 |  | 0·18 | 0·58 |  | 0·36 | 0·42 |  | 0·24 | 0·2 |  | 0·21 | 0·3 |  | 0·08 | 0·07 |  |  |  |
|  |  | (-0·32–-0·09) | (-0·22–-0·09) |  | (0·11–0·25) | (0·48–0·67) |  | (0·27–0·44) | (0·33–0·50) |  | (0·16–0·33) | (0·12–0·27) |  | (0·13–0·28) | (0·18–0·41) |  | (-0·17–0·33) | (-0·15–0·28) |  |  |  |
|  | Timor-Leste | 0·16 | 0·02 |  | 0·25 | 0·25 |  | 0·61 | 0·6 |  | 0·52 | 0·16 |  | 0·32 | 0·37 |  | 0·16 | 0·12 |  |  |  |
|  |  | (0·09–0·23) | (-0·03–0·08) |  | (0·19–0·31) | (0·17–0·34) |  | (0·53–0·70) | (0·52–0·69) |  | (0·44–0·60) | (0·16–0·16) |  | (0·21–0·43) | (0·23–0·51) |  | (-0·05–0·37) | (-0·11–0·35) |  |  |  |
|  | Zambia | 0·04 | 0·04 |  | 0·08 | 0·23 |  | 0·25 | 0·24 |  | 0·17 | 0·17 |  | 0·03 | 0·14 |  | -0·03 | 0·05 |  | 0·07 | -0·08 |
|  |  | (-0·06–0·13) | (-0·04–0·13) |  | (0·04–0·11) | (0·15–0·32) |  | (0·17–0·32) | (0·16–0·32) |  | (0·11–0·23) | (0·11–0·24) |  | (0·00–0·07) | (0·06–0·23) |  | (-0·18–0·11) | (-0·13–0·22) |  | (0·01–0·14) | (-0·16–0·00) |
|  |  |  |  |  |  |  |  |  |  |  |  |  |  |  |  |  |  |  |  |  |  |
| **Low-income** | Afghanistan | 0·13 | 0·06 |  | 0·21 | 0·05 |  | 0·59 | 0·59 |  | 0·53 | 0·01 |  | 0·18 | 0·19 |  | 0·04 | 0·01 |  |  |  |
|  |  | (0·04–0·21) | (0·02–0·09) |  | (0·11–0·30) | (0·03–0·08) |  | (0·50–0·68) | (0·50–0·68) |  | (0·44–0·62) | (-0·00–0·02) |  | (0·06–0·30) | (0·07–0·30) |  | (-0·08–0·16) | (-0·09–0·12) |  |  |  |
|  | Benin | 0·11 | 0·03 |  | 0·34 | 0·5 |  | 0·46 | 0·46 |  | 0·42 | 0·31 |  | 0·32 | 0·36 |  | 0·09 | 0·1 |  | 0·09 | 0·11 |
|  |  | (0·04–0·19) | (0·01–0·06) |  | (0·28–0·41) | (0·45–0·55) |  | (0·39–0·52) | (0·39–0·53) |  | (0·35–0·48) | (0·25–0·36) |  | (0·24–0·41) | (0·26–0·46) |  | (-0·04–0·22) | (0·00–0·20) |  | (0·05–0·13) | (0·06–0·16) |
|  | Burundi | 0·04 | -0·01 |  | 0·02 | 0·06 |  | 0·16 | 0·17 |  | 0·14 | 0·15 |  | 0·01 | 0·01 |  | 0·07 | 0·07 |  | 0·4 | 0·39 |
|  |  | (0·01–0·08) | (-0·04–0·02) |  | (0·00–0·03) | (0·04–0·08) |  | (0·12–0·21) | (0·13–0·22) |  | (0·11–0·18) | (0·11–0·20) |  | (-0·01–0·03) | (-0·04–0·06) |  | (-0·01–0·15) | (-0·01–0·15) |  | (0·34–0·46) | (0·33–0·45) |
|  | Cambodia | 0·23 | 0·1 |  | 0·38 | 0·18 |  | 0·21 | 0·19 |  | 0·24 | 0·02 |  | 0·27 | 0·41 |  | 0·15 | 0·08 |  |  |  |
|  |  | (0·12–0·33) | (0·05–0·15) |  | (0·26–0·49) | (0·12–0·24) |  | (0·21–0·21) | (0·19–0·19) |  | (0·24–0·24) | (0·02–0·02) |  | (0·10–0·44) | (0·23–0·58) |  | (-0·03–0·32) | (-0·06–0·22) |  |  |  |
|  | Chad | 0·17 | 0 |  | 0·22 | 0·41 |  | 0·54 | 0·54 |  | 0·5 | 0·17 |  | 0·35 | 0·28 |  | 0·44 | 0·66 |  | -0·06 | 0·03 |
|  |  | (0·17–0·17) | (0·00–0·00) |  | (0·12–0·32) | (0·33–0·49) |  | (0·44–0·65) | (0·43–0·64) |  | (0·40–0·61) | (0·11–0·23) |  | (0·19–0·52) | (0·12–0·44) |  | (0·26–0·62) | (0·41–0·90) |  | (-0·17–0·05) | (-0·08–0·14) |
|  | Ethiopia | 0·17 | -0·04 |  | 0·25 | 0·58 |  | 0·44 | 0·38 |  | 0·57 | 0·2 |  | 0·26 | 0·4 |  | 0·06 | 0·08 |  |  |  |
|  |  | (0·09–0·26) | (-0·10–0·01) |  | (0·19–0·31) | (0·48–0·68) |  | (0·44–0·44) | (0·38–0·38) |  | (0·48–0·67) | (0·20–0·20) |  | (0·14–0·38) | (0·26–0·55) |  | (-0·07–0·20) | (-0·03–0·19) |  |  |  |
|  | Guinea | 0 | -0·07 |  | 0·11 | 0·17 |  | 0·26 | 0·31 |  | 0·25 | 0·21 |  | 0·16 | 0·44 |  | -0·13 | -0·21 |  |  |  |
|  |  | (-0·08–0·08) | (-0·12–-0·03) |  | (0·07–0·15) | (0·06–0·28) |  | (0·18–0·33) | (0·23–0·39) |  | (0·18–0·32) | (0·10–0·31) |  | (0·10–0·22) | (0·32–0·56) |  | (-0·36–0·10) | (-0·43–0·01) |  |  |  |
|  | Haiti | 0·22 | 0·14 |  | 0·3 | 0·41 |  | 0·55 | 0·54 |  | 0·49 | 0·02 |  | 0·2 | 0·17 |  | 0·11 | 0·09 |  | -0·01 | -0·04 |
|  |  | (0·09–0·35) | (0·09–0·20) |  | (0·21–0·39) | (0·33–0·48) |  | (0·43–0·67) | (0·42–0·66) |  | (0·38–0·61) | (0·02–0·02) |  | (0·07–0·33) | (0·00–0·33) |  | (-0·05–0·27) | (-0·03–0·21) |  | (-0·04–0·03) | (-0·10–0·01) |
|  | Malawi | 0·01 | 0·02 |  | 0·03 | 0·06 |  | 0·08 | 0·09 |  | 0·06 | 0·08 |  | 0·02 | 0·09 |  | 0·04 | 0·05 |  | 0·28 | 0·25 |
|  |  | (-0·04–0·06) | (-0·03–0·07) |  | (0·01–0·05) | (0·03–0·09) |  | (0·05–0·12) | (0·05–0·12) |  | (0·03–0·09) | (0·04–0·12) |  | (-0·01–0·05) | (0·03–0·15) |  | (-0·04–0·13) | (-0·04–0·13) |  | (0·23–0·33) | (0·20–0·31) |
|  | Mali | -0·07 | -0·13 |  | 0·17 | 0·45 |  | 0·42 | 0·48 |  | 0·38 | 0·01 |  | 0·04 | 0·06 |  | 0·13 | -0·03 |  |  |  |
|  |  | (-0·14–0·00) | (-0·19–-0·08) |  | (0·09–0·25) | (0·37–0·53) |  | (0·32–0·53) | (0·48–0·48) |  | (0·29–0·48) | (0·01–0·01) |  | (-0·02–0·09) | (-0·05–0·16) |  | (-0·18–0·44) | (-0·23–0·17) |  |  |  |
|  | Nepal | 0·17 | 0·04 |  | 0·01 | 0·06 |  | 0·13 | 0·14 |  | 0·1 | 0·04 |  | 0·03 | 0·14 |  | 0·21 | 0·15 |  |  |  |
|  |  | (0·12–0·22) | (-0·01–0·09) |  | (0·00–0·02) | (-0·00–0·12) |  | (0·09–0·17) | (0·10–0·17) |  | (0·07–0·13) | (0·00–0·08) |  | (0·00–0·05) | (0·03–0·26) |  | (0·07–0·35) | (0·03–0·26) |  |  |  |
|  | Rwanda | 0·14 | 0·21 |  | 0·07 | 0·43 |  | 0·62 | 0·61 |  | 0·34 | 0·28 |  | 0·1 | 0·25 |  | 0·14 | 0·05 |  |  |  |
|  |  | (0·08–0·20) | (0·10–0·32) |  | (0·04–0·10) | (0·37–0·49) |  | (0·54–0·70) | (0·53–0·69) |  | (0·29–0·40) | (0·21–0·34) |  | (0·05–0·15) | (0·15–0·35) |  | (0·03–0·25) | (-0·06–0·17) |  |  |  |
|  | Senegal | 0·04 | 0·01 |  | 0·05 | 0·08 |  | 0·08 | 0·07 |  | 0·07 | 0 |  | 0·12 | 0·13 |  | 0·16 | 0·14 |  | 0·06 | -0·02 |
|  |  | (-0·04–0·12) | (0·01–0·01) |  | (-0·05–0·14) | (0·04–0·13) |  | (0·01–0·16) | (0·01–0·13) |  | (-0·02–0·15) | (-0·01–0·00) |  | (0·00–0·24) | (0·03–0·23) |  | (0·01–0·31) | (0·04–0·23) |  | (0·02–0·10) | (-0·09–0·05) |
|  | Tajikistan | 0·15 | 0·09 |  | 0·11 | 0·43 |  | 0·08 | 0·16 |  | 0·04 | 0·25 |  | 0·02 | -0·07 |  | 0·04 | 0·01 |  |  |  |
|  |  | (0·03–0·26) | (0·02–0·16) |  | (0·05–0·17) | (0·33–0·53) |  | (0·03–0·13) | (0·09–0·24) |  | (0·01–0·08) | (0·13–0·37) |  | (-0·05–0·09) | (-0·20–0·06) |  | (-0·15–0·23) | (-0·16–0·18) |  |  |  |
|  | Tanzania | 0·05 | 0·06 |  | 0·02 | 0·33 |  | 0·49 | 0·49 |  | 0·44 | 0·23 |  | 0·08 | 0·25 |  | 0·05 | 0·09 |  | 0·21 | 0·19 |
|  |  | (-0·02–0·13) | (-0·00–0·12) |  | (-0·01–0·04) | (0·27–0·39) |  | (0·39–0·59) | (0·39–0·59) |  | (0·34–0·53) | (0·16–0·29) |  | (0·04–0·12) | (0·14–0·36) |  | (-0·10–0·21) | (-0·06–0·25) |  | (0·11–0·30) | (0·09–0·29) |
|  | Uganda | -0·02 | 0·02 |  | 0·01 | 0·2 |  | 0·27 | 0·28 |  | 0·21 | 0 |  | 0 | 0·07 |  | -0·06 | -0·01 |  | 0·18 | 0·17 |
|  |  | (-0·08–0·03) | (-0·02–0·07) |  | (-0·01–0·04) | (0·16–0·24) |  | (0·22–0·33) | (0·22–0·33) |  | (0·16–0·27) | (-0·04–0·05) |  | (-0·04–0·04) | (-0·01–0·15) |  | (-0·14–0·02) | (-0·09–0·08) |  | (0·14–0·23) | (0·11–0·23) |
|  | Zimbabwe | 0·17 | 0·21 |  | 0·12 | 0·38 |  | 0·33 | 0·34 |  | 0·2 | 0·32 |  | 0·07 | 0·17 |  | 0·18 | 0·22 |  |  |  |
|  |  | (0·06–0·28) | (0·11–0·32) |  | (0·04–0·21) | (0·25–0·51) |  | (0·22–0·44) | (0·23–0·45) |  | (0·11–0·29) | (0·20–0·43) |  | (-0·07–0·21) | (-0·02–0·36) |  | (-0·03–0·39) | (0·03–0·42) |  |  |  |

**Notes:**

SII — slope index of inequality, Diff — difference

SII > 0 represents higher inequality.

Dark green indicates smaller inequality, red indicates higher inequality, grey indicates data not available.

**Appendix 15. Erreygers’ concentration index of RMNCH services**

| **Income group** | **Countries** | **Family Planning** | |  | **Antenatal care** | |  | **Delivery care** | |  | **Postnatal care** | |  | **Immunisation** | |  | **Diarrhoea treatment** | |  | **Use of insecticide-treated nets** | |
| --- | --- | --- | --- | --- | --- | --- | --- | --- | --- | --- | --- | --- | --- | --- | --- | --- | --- | --- | --- | --- | --- |
|  |  | **Contact** | **Quality** |  | **Contact** | **Quality** |  | **Contact** | **Crude** |  | **Contact** | **Quality** |  | **Contact** | **Quality** |  | **Contact** | **Quality** |  | **Contact** | **Crude** |
|  |  | **CI (95% CI)** | |  | **CI (95% CI)** | |  | **CI (95% CI)** | |  | **CI (95% CI)** | |  | **CI (95% CI)** | |  | **CI (95% CI)** | |  | **CI (95% CI)** | |
| **Upper-middle-income** | Albania | 0·15 | 0·00 |  | 0·08 | 0·23 |  | 0·01 | -0·01 |  | 0 | 0·04 |  | -0·03 | -0·12 |  | -0·02 | 0·16 |  |  |  |
|  |  | (0·10–0·20) | (-0·00–0·01) |  | (0·03–0·12) | (0·15–0·31) |  | (-0·00–0·01) | (-0·03–0·01) |  | (-0·00–0·00) | (-0·04–0·12) |  | (-0·12–0·05) | (-0·28–0·03) |  | (-0·26–0·22) | (-0·06–0·38) |  |  |  |
|  | Colombia | 0·11 | 0·07 |  | 0·06 | 0·11 |  | 0·08 | 0·08 |  |  |  |  |  |  |  |  |  |  |  |  |
|  |  | (0·08–0·14) | (0·03–0·11) |  | (0·03–0·08) | (0·08–0·15) |  | (0·06–0·10) | (0·06–0·11) |  |  |  |  |  |  |  |  |  |  |  |  |
|  | Jordan | -0·03 | -0·04 |  | 0·02 | 0·08 |  | 0 | 0·01 |  | 0 | -0·01 |  | 0·02 | -0·01 |  | 0·07 | 0·1 |  |  |  |
|  |  | (-0·08–0·02) | (-0·07–-0·02) |  | (0·00–0·03) | (0·03–0·13) |  | (-0·00–0·01) | (-0·00–0·02) |  | (-0·00–0·00) | (-0·05–0·03) |  | (-0·03–0·07) | (-0·09–0·06) |  | (-0·05–0·19) | (-0·01–0·21) |  |  |  |
|  | Maldives | -0·01 | 0·02 |  | 0 | 0·06 |  | -0·01 | -0·04 |  | 0 | 0·02 |  | -0·01 | -0·01 |  | -0·06 | 0·14 |  |  |  |
|  |  | (-0·05–0·03) | (-0·01–0·04) |  | (-0·07–0·07) | (-0·01–0·14) |  | (-0·03–0·01) | (-0·08–0·00) |  | (-0·00–0·00) | (-0·07–0·12) |  | (-0·09–0·08) | (-0·13–0·12) |  | (-0·26–0·14) | (-0·12–0·39) |  |  |  |
|  | South Africa | -0·02 | -0·02 |  | 0 | 0·05 |  | 0·04 | 0·05 |  | 0·01 | 0·05 |  | 0·03 | 0 |  | 0·18 | 0·22 |  |  |  |
|  |  | (-0·07–0·03) | (-0·06–0·02) |  | (-0·04–0·04) | (-0·02–0·11) |  | (0·02–0·06) | (0·02–0·07) |  | (-0·01–0·02) | (-0·02–0·11) |  | (-0·05–0·10) | (-0·11–0·11) |  | (0·05–0·31) | (0·08–0·37) |  |  |  |
|  |  |  |  |  |  |  |  |  |  |  |  |  |  |  |  |  |  |  |  |  |  |
|  | Angola | 0·37 | 0·11 |  | 0·34 | 0·56 |  | 0·58 | 0·61 |  | 0·52 | 0·14 |  | 0·41 | 0·37 |  | 0·17 | 0·2 |  | 0·04 | 0·05 |
|  |  | (0·32–0·41) | (0·07–0·14) |  | (0·31–0·38) | (0·52–0·59) |  | (0·54–0·62) | (0·57–0·64) |  | (0·48–0·56) | (0·11–0·17) |  | (0·36–0·47) | (0·31–0·42) |  | (0·08–0·25) | (0·11–0·28) |  | (-0·01–0·09) | (0·00–0·10) |
| **Lower-middle-income** | Armenia | 0·22 | 0·03 |  | 0·02 | 0·11 |  | 0 | 0 |  | 0 | -0·01 |  | 0·01 | -0·09 |  | -0·11 | 0 |  |  |  |
|  |  | (0·17–0·27) | (0·01–0·05) |  | (-0·01–0·05) | (0·02–0·20) |  | (-0·00–0·00) | (-0·00–0·01) |  | (0·00–0·00) | (-0·08–0·06) |  | (-0·01–0·03) | (-0·19–0·02) |  | (-0·42–0·20) | (-0·22–0·23) |  |  |  |
|  | Bangladesh | -0·11 |  |  | 0·42 | 0·33 |  | 0·44 | 0·43 |  | 0·44 | 0·03 |  | 0·04 | 0·18 |  | 0·15 | 0·08 |  |  |  |
|  |  | (-0·14–-0·07) |  |  | (0·38–0·45) | (0·29–0·36) |  | (0·40–0·48) | (0·39–0·47) |  | (0·39–0·48) | (0·02–0·04) |  | (0·01–0·06) | (0·12–0·24) |  | (-0·03–0·33) | (-0·11–0·28) |  |  |  |
|  | Cameroon | 0·06 | 0·05 |  | 0·23 | 0·49 |  | 0·53 | 0·55 |  | 0·5 | 0·29 |  | 0·19 | 0·28 |  | 0·05 | 0·11 |  | -0·01 | -0·07 |
|  |  | (0·02–0·11) | (0·02–0·07) |  | (0·19–0·27) | (0·45–0·53) |  | (0·49–0·58) | (0·50–0·59) |  | (0·46–0·55) | (0·25–0·33) |  | (0·14–0·24) | (0·22–0·35) |  | (-0·04–0·13) | (0·04–0·17) |  | (-0·05–0·02) | (-0·11–-0·03) |
|  | Ghana | 0·08 | -0·03 |  | 0·06 | 0·21 |  | 0·41 | 0·42 |  | 0·19 | 0·08 |  | 0·02 | 0·04 |  | -0·04 | 0·03 |  | -0·06 | -0·24 |
|  |  | (0·01–0·14) | (-0·06–0·01) |  | (0·03–0·09) | (0·15–0·27) |  | (0·36–0·46) | (0·36–0·47) |  | (0·13–0·24) | (0·03–0·13) |  | (-0·01–0·04) | (-0·03–0·11) |  | (-0·15–0·07) | (-0·09–0·16) |  | (-0·11–-0·02) | (-0·30–-0·18) |
|  | Guatemala | -0·04 | 0·07 |  | 0·1 | 0·35 |  | 0·48 | 0·48 |  | 0·07 | -0·01 |  | 0·01 | 0·12 |  | -0·01 | 0·08 |  |  |  |
|  |  | (-0·07–-0·02) | (0·05–0·09) |  | (0·08–0·12) | (0·31–0·39) |  | (0·45–0·51) | (0·44–0·51) |  | (0·06–0·08) | (-0·02–-0·00) |  | (-0·01–0·03) | (0·06–0·17) |  | (-0·07–0·04) | (0·02–0·14) |  |  |  |
|  | India | 0·04 | 0 |  | 0·28 | 0·38 |  | 0·24 | 0·27 |  | 0·22 | 0·08 |  | 0·07 | 0·14 |  | 0·09 | 0·14 |  |  |  |
|  |  | (0·02–0·06) | (-0·00–0·01) |  | (0·28–0·29) | (0·37–0·39) |  | (0·23–0·25) | (0·26–0·28) |  | (0·21–0·22) | (0·07–0·08) |  | (0·06–0·08) | (0·13–0·16) |  | (0·07–0·10) | (0·11–0·16) |  |  |  |
|  | Indonesia | 0·07 | 0·02 |  | 0·06 | 0·19 |  | 0·17 | 0·3 |  | 0·17 | 0·03 |  | 0·07 | 0·12 |  | 0 | 0 |  |  |  |
|  |  | (0·05–0·09) | (0·01–0·03) |  | (0·04–0·07) | (0·16–0·22) |  | (0·14–0·19) | (0·27–0·33) |  | (0·14–0·19) | (0·02–0·05) |  | (0·04–0·10) | (0·08–0·17) |  | (-0·05–0·05) | (-0·06–0·05) |  |  |  |
|  | Kenya | 0 | 0·09 |  | 0·07 | 0·25 |  | 0·49 | 0·5 |  | 0·39 | 0·08 |  | 0·03 | 0·15 |  | -0·04 | -0·03 |  | 0·15 | 0·2 |
|  |  | (-0·04–0·04) | (0·06–0·13) |  | (0·06–0·09) | (0·22–0·28) |  | (0·46–0·51) | (0·47–0·52) |  | (0·37–0·41) | (0·06–0·10) |  | (0·01–0·05) | (0·11–0·19) |  | (-0·10–0·02) | (-0·08–0·03) |  | (0·12–0·18) | (0·17–0·24) |
|  | Lesotho | -0·01 | 0·02 |  | 0·05 | 0·21 |  | 0·25 | 0·27 |  | 0·08 | 0·03 |  | 0·03 | 0·03 |  | -0·03 | -0·19 |  |  |  |
|  |  | (-0·06–0·04) | (-0·02–0·05) |  | (0·02–0·08) | (0·15–0·28) |  | (0·21–0·29) | (0·23–0·32) |  | (0·06–0·11) | (-0·01–0·06) |  | (0·01–0·05) | (-0·06–0·11) |  | (-0·20–0·13) | (-0·34–-0·05) |  |  |  |
|  | Myanmar | 0·16 | 0·04 |  | 0·23 | 0·41 |  | 0·45 | 0·47 |  | 0·4 | 0 |  | 0·13 | 0·29 |  | 0·11 | 0 |  |  |  |
|  |  | (0·12–0·21) | (0·01–0·06) |  | (0·18–0·29) | (0·36–0·47) |  | (0·39–0·51) | (0·42–0·52) |  | (0·34–0·45) | (-0·00–0·01) |  | (0·07–0·20) | (0·20–0·38) |  | (-0·01–0·22) | (-0·11–0·12) |  |  |  |
|  | Nigeria | 0·03 | 0·06 |  | 0·4 | 0·39 |  | 0·6 | 0·54 |  | 0·59 | 0·16 |  | 0·43 | 0·44 |  | 0·09 | 0·15 |  | -0·14 | -0·2 |
|  |  | (-0·00–0·06) | (0·04–0·07) |  | (0·37–0·43) | (0·36–0·42) |  | (0·58–0·62) | (0·52–0·56) |  | (0·56–0·61) | (0·15–0·18) |  | (0·40–0·47) | (0·40–0·48) |  | (0·05–0·14) | (0·11–0·20) |  | (-0·17–-0·11) | (-0·23–-0·17) |
|  | Pakistan | 0·05 | 0 |  | 0·24 | 0·46 |  | 0·36 | 0·37 |  | 0·34 | 0·14 |  | 0·21 | 0·34 |  | 0·09 | 0 |  |  |  |
|  |  | (0·00–0·09) | (-0·01–0·01) |  | (0·20–0·27) | (0·41–0·51) |  | (0·31–0·41) | (0·33–0·42) |  | (0·29–0·38) | (0·11–0·17) |  | (0·15–0·27) | (0·27–0·40) |  | (0·02–0·16) | (-0·07–0·08) |  |  |  |
|  | Papua New Guinea | 0·18 | 0·05 |  | 0·27 | 0·2 |  | 0·46 | 0·47 |  | 0·42 | 0·25 |  | 0·37 | 0·29 |  | 0·11 | 0·12 |  |  |  |
|  |  | (0·12–0·23) | (0·02–0·07) |  | (0·22–0·32) | (0·16–0·25) |  | (0·41–0·51) | (0·41–0·52) |  | (0·37–0·47) | (0·19–0·30) |  | (0·29–0·44) | (0·23–0·36) |  | (0·01–0·21) | (0·03–0·21) |  |  |  |
|  | Philippines | -0·18 | -0·13 |  | 0·1 | 0·38 |  | 0·24 | 0·28 |  | 0·16 | 0·13 |  | 0·13 | 0·18 |  | 0 | 0·01 |  |  |  |
|  |  | (-0·23–-0·13) | (-0·16–-0·10) |  | (0·07–0·13) | (0·33–0·42) |  | (0·20–0·28) | (0·24–0·32) |  | (0·12–0·20) | (0·08–0·18) |  | (0·09–0·17) | (0·12–0·25) |  | (-0·12–0·13) | (-0·10–0·12) |  |  |  |
|  | Timor-Leste | 0·07 | 0 |  | 0·18 | 0·32 |  | 0·51 | 0·57 |  | 0·42 | 0·18 |  | 0·21 | 0·18 |  | 0·15 | 0·11 |  |  |  |
|  |  | (0·02–0·11) | (-0·03–0·04) |  | (0·14–0·21) | (0·27–0·37) |  | (0·47–0·55) | (0·53–0·61) |  | (0·38–0·46) | (0·14–0·21) |  | (0·15–0·27) | (0·11–0·26) |  | (0·04–0·25) | (-0·00–0·22) |  |  |  |
|  | Zambia | -0·1 | -0·01 |  | 0·06 | 0·15 |  | 0·21 | 0·21 |  | 0·14 | 0·22 |  | 0·02 | 0·07 |  | -0·11 | 0·06 |  | -0·01 | -0·06 |
|  |  | (-0·14–-0·05) | (-0·05–0·03) |  | (0·04–0·08) | (0·11–0·20) |  | (0·18–0·25) | (0·17–0·24) |  | (0·11–0·17) | (0·19–0·26) |  | (-0·00–0·04) | (0·03–0·11) |  | (-0·19–-0·03) | (-0·01–0·13) |  | (-0·04–0·02) | (-0·11–-0·02) |
|  |  |  |  |  |  |  |  |  |  |  |  |  |  |  |  |  |  |  |  |  |  |
| **Low-income** | Afghanistan | 0·05 | 0·01 |  | 0·19 | 0·07 |  | 0·47 | 0·47 |  | 0·42 | 0·02 |  | 0·15 | 0·15 |  | 0·04 | 0 |  |  |  |
|  |  | (-0·00–0·10) | (-0·01–0·03) |  | (0·15–0·23) | (0·05–0·10) |  | (0·44–0·50) | (0·44–0·51) |  | (0·38–0·45) | (0·01–0·04) |  | (0·10–0·20) | (0·09–0·21) |  | (-0·01–0·09) | (-0·06–0·06) |  |  |  |
|  | Benin | 0·06 | 0·02 |  | 0·22 | 0·33 |  | 0·29 | 0·29 |  | 0·26 | 0·18 |  | 0·2 | 0·22 |  | 0·07 | 0·09 |  | 0·05 | 0·08 |
|  |  | (0·02–0·10) | (0·01–0·04) |  | (0·18–0·26) | (0·30–0·36) |  | (0·25–0·33) | (0·25–0·33) |  | (0·23–0·30) | (0·15–0·22) |  | (0·15–0·24) | (0·17–0·27) |  | (-0·00–0·15) | (0·02–0·15) |  | (0·03–0·08) | (0·05–0·11) |
|  | Burundi | -0·01 | -0·01 |  | 0·01 | 0·07 |  | 0·11 | 0·11 |  | 0·1 | 0·12 |  | 0 | 0 |  | 0·05 | 0·02 |  | 0·26 | 0·25 |
|  |  | (-0·03–0·01) | (-0·02–0·01) |  | (0·00–0·02) | (0·05–0·09) |  | (0·09–0·13) | (0·09–0·14) |  | (0·07–0·12) | (0·09–0·15) |  | (-0·01–0·02) | (-0·03–0·03) |  | (0·00–0·10) | (-0·03–0·07) |  | (0·23–0·29) | (0·22–0·28) |
|  | Cambodia | 0 | -0·04 |  | 0·07 | 0·15 |  | 0·15 | 0·19 |  | 0·15 | 0·13 |  | 0·1 | 0·27 |  | -0·04 | -0·1 |  |  |  |
|  |  | (-0·04–0·04) | (-0·07–-0·02) |  | (0·05–0·09) | (0·09–0·20) |  | (0·11–0·19) | (0·15–0·24) |  | (0·11–0·19) | (0·08–0·18) |  | (0·07–0·13) | (0·21–0·32) |  | (-0·13–0·05) | (-0·21–0·01) |  |  |  |
|  | Chad | 0·11 | 0·02 |  | 0·14 | 0·16 |  | 0·27 | 0·27 |  | 0·26 | 0 |  | 0·13 | 0·09 |  | 0·1 | 0·09 |  | 0·06 | 0·07 |
|  |  | (0·06–0·15) | (0·00–0·03) |  | (0·10–0·18) | (0·14–0·19) |  | (0·23–0·31) | (0·23–0·30) |  | (0·21–0·30) | (0·00–0·01) |  | (0·07–0·19) | (0·04–0·14) |  | (0·04–0·16) | (0·04–0·13) |  | (0·04–0·07) | (0·04–0·10) |
|  | Ethiopia | 0·15 | 0·1 |  | 0·28 | 0·21 |  | 0·41 | 0·41 |  | 0·42 | 0·06 |  | 0·19 | 0·26 |  | 0·13 | 0·07 |  |  |  |
|  |  | (0·10–0·20) | (0·07–0·13) |  | (0·22–0·33) | (0·18–0·24) |  | (0·36–0·45) | (0·36–0·45) |  | (0·37–0·46) | (0·04–0·07) |  | (0·11–0·27) | (0·18–0·34) |  | (0·03–0·22) | (-0·00–0·15) |  |  |  |
|  | Guinea | 0·16 | 0·04 |  | 0·18 | 0·35 |  | 0·54 | 0·51 |  | 0·45 | 0·2 |  | 0·28 | 0·19 |  | 0·25 | 0·27 |  | -0·09 | 0 |
|  |  | (0·09–0·22) | (0·02–0·06) |  | (0·13–0·23) | (0·31–0·39) |  | (0·50–0·58) | (0·47–0·55) |  | (0·40–0·49) | (0·16–0·23) |  | (0·20–0·36) | (0·12–0·27) |  | (0·16–0·33) | (0·18–0·36) |  | (-0·14–-0·04) | (-0·05–0·04) |
|  | Haiti | 0·07 | -0·02 |  | 0·13 | 0·36 |  | 0·51 | 0·48 |  | 0·37 | 0·13 |  | 0·15 | 0·26 |  | 0·14 | 0·11 |  |  |  |
|  |  | (0·03–0·11) | (-0·05–0·00) |  | (0·10–0·16) | (0·31–0·41) |  | (0·47–0·54) | (0·44–0·52) |  | (0·32–0·42) | (0·10–0·15) |  | (0·08–0·22) | (0·17–0·34) |  | (0·07–0·22) | (0·05–0·18) |  |  |  |
|  | Malawi | -0·02 | -0·01 |  | 0·02 | 0·07 |  | 0·06 | 0·06 |  | 0·04 | 0·08 |  | 0 | 0·02 |  | -0·01 | -0·01 |  | 0·16 | 0·16 |
|  |  | (-0·05–0·01) | (-0·04–0·02) |  | (0·01–0·04) | (0·05–0·10) |  | (0·04–0·08) | (0·04–0·08) |  | (0·03–0·06) | (0·06–0·11) |  | (-0·01–0·02) | (-0·01–0·06) |  | (-0·06–0·04) | (-0·06–0·04) |  | (0·13–0·18) | (0·13–0·19) |
|  | Mali | 0·15 | 0·09 |  | 0·21 | 0·39 |  | 0·4 | 0·4 |  | 0·36 | 0·15 |  | 0·15 | 0·15 |  | 0·06 | 0·02 |  | -0·03 | -0·08 |
|  |  | (0·08–0·21) | (0·07–0·12) |  | (0·16–0·25) | (0·35–0·43) |  | (0·34–0·46) | (0·35–0·46) |  | (0·31–0·42) | (0·12–0·18) |  | (0·09–0·21) | (0·07–0·22) |  | (-0·02–0·14) | (-0·04–0·07) |  | (-0·04–-0·01) | (-0·11–-0·05) |
|  | Nepal | -0·02 | -0·08 |  | 0·13 | 0·37 |  | 0·37 | 0·37 |  | 0·31 | 0·1 |  | 0·02 | 0·03 |  | 0·05 | -0·03 |  |  |  |
|  |  | (-0·07–0·02) | (-0·11–-0·04) |  | (0·08–0·17) | (0·32–0·41) |  | (0·31–0·42) | (0·32–0·43) |  | (0·26–0·36) | (0·07–0·13) |  | (-0·01–0·05) | (-0·03–0·09) |  | (-0·15–0·26) | (-0·16–0·11) |  |  |  |
|  | Rwanda | 0·06 | -0·01 |  | 0 | 0·06 |  | 0·09 | 0·09 |  | 0·07 | 0·01 |  | 0·01 | 0·09 |  | 0·13 | 0·09 |  |  |  |
|  |  | (0·03–0·09) | (-0·04–0·02) |  | (-0·00–0·01) | (0·03–0·09) |  | (0·07–0·11) | (0·07–0·11) |  | (0·05–0·08) | (-0·01–0·04) |  | (-0·00–0·03) | (0·03–0·15) |  | (0·05–0·21) | (0·02–0·16) |  |  |  |
|  | Senegal | 0·1 | 0·22 |  | 0·05 | 0·34 |  | 0·46 | 0·45 |  | 0·22 | 0·22 |  | 0·06 | 0·17 |  | 0·11 | 0·02 |  |  |  |
|  |  | (0·07–0·13) | (0·16–0·27) |  | (0·03–0·06) | (0·31–0·38) |  | (0·42–0·50) | (0·41–0·49) |  | (0·19–0·26) | (0·18–0·26) |  | (0·03–0·08) | (0·12–0·21) |  | (0·05–0·18) | (-0·03–0·08) |  |  |  |
|  | Tajikistan | 0·12 | 0·06 |  | 0·07 | 0·24 |  | 0·05 | 0·11 |  | 0·03 | 0·15 |  | -0·02 | -0·08 |  | 0·05 | 0·05 |  |  |  |
|  |  | (0·08–0·17) | (0·03–0·09) |  | (0·04–0·10) | (0·20–0·29) |  | (0·02–0·08) | (0·07–0·15) |  | (0·01–0·05) | (0·10–0·21) |  | (-0·05–0·02) | (-0·14–-0·02) |  | (-0·04–0·14) | (-0·03–0·13) |  |  |  |
|  | Tanzania | 0·04 | 0·02 |  | 0·01 | 0·32 |  | 0·41 | 0·41 |  | 0·34 | 0·23 |  | 0·05 | 0·17 |  | 0·03 | 0·06 |  | 0·16 | 0·2 |
|  |  | (0·00–0·08) | (-0·01–0·06) |  | (-0·00–0·03) | (0·29–0·36) |  | (0·37–0·45) | (0·37–0·45) |  | (0·30–0·38) | (0·20–0·27) |  | (0·03–0·07) | (0·11–0·23) |  | (-0·04–0·11) | (-0·02–0·14) |  | (0·12–0·21) | (0·15–0·25) |
|  | Uganda | -0·04 | 0·02 |  | 0·01 | 0·19 |  | 0·23 | 0·23 |  | 0·18 | 0·05 |  | 0 | 0·05 |  | -0·03 | 0·02 |  | 0·11 | 0·13 |
|  |  | (-0·07–-0·00) | (-0·00–0·05) |  | (-0·01–0·02) | (0·17–0·22) |  | (0·20–0·26) | (0·20–0·27) |  | (0·15–0·21) | (0·02–0·08) |  | (-0·02–0·02) | (0·00–0·09) |  | (-0·08–0·02) | (-0·03–0·08) |  | (0·09–0·14) | (0·10–0·15) |
|  | Zimbabwe | 0·03 | 0·05 |  | 0·07 | 0·2 |  | 0·24 | 0·24 |  | 0·14 | 0·19 |  | 0·07 | 0·1 |  | 0·06 | 0·1 |  |  |  |
|  |  | (-0·02–0·07) | (0·01–0·10) |  | (0·03–0·10) | (0·15–0·25) |  | (0·20–0·28) | (0·20–0·29) |  | (0·10–0·18) | (0·14–0·23) |  | (0·02–0·12) | (0·03–0·17) |  | (-0·03–0·15) | (0·01–0·18) |  |  |  |

**Notes:**

CI — Erreygers’ concentration index, Diff — difference

CI > 0 represents higher inequality.

Dark green indicates smaller inequality, red indicates higher inequality, grey indicates data not available

1. World Bank Country and Lending Groups: historical classification by income in XLS format. Source: <https://datahelpdesk.worldbank.org/knowledgebase/articles/906519-world-bank-country-and-lending-groups> [↑](#footnote-ref-1)
2. We only included malaria high transmission countries. Source: <https://www.who.int/publications/i/item/9789241565721> [↑](#footnote-ref-2)
3. <https://www.measureevaluation.org/prh/rh_indicators/family-planning/method-choice/method-information-index> [↑](#footnote-ref-3)
4. https://www.who.int/Immunisation/policy/Immunisation_routine_table2.pdf [↑](#footnote-ref-4)
5. https://apps.who.int/iris/bitstream/handle/10665/44471/9789241548083_eng.pdf?sequence=1 [↑](#footnote-ref-5)
6. <https://www.who.int/publications/i/item/9789241565721> [↑](#footnote-ref-6)
7. Amouzou A, Leslie HH, Ram M, et al. Advances in the measurement of coverage for RMNCH and nutrition: from contact to effective coverage. BMJ Glob Heal 2019; 4: 114–124. [↑](#footnote-ref-7)
8. <https://www.measureevaluation.org/prh/rh_indicators/family-planning/method-choice/method-information-index> [↑](#footnote-ref-8)
9. Arsenault C, Jordan K, Lee D, et al. Equity in antenatal care quality: an analysis of 91 national household surveys. *Lancet Glob Heal* 2018; 6: e1186–e1195.

   [↑](#footnote-ref-9)
10. Nguhiu PK, Barasa EW, Chuma J. Determining the effective coverage of maternal and child health services in Kenya, using demographic and health survey data sets: tracking progress towards universal health coverage. Trop Med Int Heal 2017; 22: 442–453. [↑](#footnote-ref-10)
